# Design, development and pilot of a realistic virtual reality application to analyse quick directional change in sport: Avatar cutting scenario with alterable parameters

**DOI:** 10.1371/journal.pone.0324941

**Published:** 2025-06-24

**Authors:** Hannah K. M. Tang, Mark J. Lake, Richard J. Foster, Frederic A. Bezombes

**Affiliations:** 1 School of Engineering, LJMU, Liverpool, United Kingdom; 2 Research Institute for Sport and Exercise Sciences, LJMU, Liverpool, United Kingdom; Ningbo University, CHINA

## Abstract

Injuries during quick directional change (cutting) are common, particularly to the anterior cruciate ligament of the knee. Cutting is subsequently a focus in research and clinical practices. However, it is usually assessed in situations with low ecological validity. As a solution, virtual reality (VR) has been used to replicate sporting scenarios. The current paper details the design, development, and piloting of a VR application based on an unanticipated-cutting scenario using an avatar-opponent blocking manoeuvre. The VR environment is highly realistic, avatar approach is instigated by the movement of the headset user, and simple input alters the avatar’s movements and spatiotemporal demands of the cutting task. Piloting occurred in two stages: the first involved three participants and focused on initial system testing and parameter optimisation, the second focused on a pilot case study with a female participant of typical population. The case study evaluated the effects of using different visual cutting cues on frontal plane knee kinematics and kinetics linked to injury risk. Twenty-five successful, unanticipated, 90⁰ cutting manoeuvres to the left were analysed in three conditions: 1) physical world with arrows, 2) VR environment emulation with VR arrows, 3) VR environment emulation with VR avatar opponent. Mean knee angle during the arrow conditions, both physical and VR, presented abduction angles (−8.82° ± 1.44; −2.66° ± 0.90), yet cutting around the avatar opponent presented knee adduction angles (9.78° ± 0.44). During the 50 ms prior to heel strike, pelvis velocity was lowest and cutting foot velocity highest in the avatar condition compared to arrow conditions. This indicated the VR environment acts as a suitable control and that cutting strategy was adapted approaching the avatar, displaying more caution on approach. The avatar-based application has the potential to improve the ecological validity of cutting assessment and make a VR application more accessible to researchers and clinicians, with further development.

## 1. Introduction

Quick directional change (cutting) is required in multidirectional sports and is associated with injury [1]. These manoeuvres involve a sprint approach followed by a sudden deceleration, change-of-direction foot plant, and reacceleration in a new direction [2]. This allows an athlete to move around an opponent [3] and is vital for performance [4]. However, the spatiotemporal requirements of the task mean the pivot leg can be subjected to excessive mechanical overloading which can shatter overall tissue integrity [5]. As cutting is common in team sports, cutting injuries are prevalent, especially the rupture of ligaments in the knee (anterior cruciate ligament injury (ACLI)) [6,7] and ankle sprain (anterior talofibular ligament sprain) [8]. Consequently, a lot of research in the analysis of biomechanics and athlete training focuses on cutting injury mechanics, screening tools, and return to sport (RTS) [4,9–15].

A limitation to cutting analysis is that assessment environments and tasks are not representative of real sports and may result in inauthentic movements [16]. In 2022, Di Paolo et al. [17] analysed cutting tasks in a laboratory compared to on-field agility exercises. The kinematics differed in each plane. Di Paolo et al. [17] suggested that testing players in an ecological environment, such as on-field, may improve ACLI-prevention programmes. However, there are limitations to both: laboratories and clinics are restrictive regarding space and struggle to emulate the nuances of a realistic cutting task [17], whereas on-field data collection equipment is considered insufficient primarily due to lack of accuracy and precision [18,19]. Inaccessibility to specific settings is also a limitation [20]. Therefore, to optimise internal and external validity, attempts must be made to improve the ecological validity of laboratory and clinical settings [21].

Virtual reality (VR) environments are formed of easily manipulated, dynamic three-dimensional (3D) stimuli [22], providing management over environmental visualisation in sporting contexts [23,24]. A review of research utilising VR tailored to athlete ACLI prevention and rehabilitation, with an overview on cutting, can be found in the work of Soltanabadi et al. [23]. VR has been applied in the analysis of cutting manoeuvres, both to replicate sporting environments and for the assessment of biomechanical movement patterns [25–27]. There are three main contributing reasons for the increased use of VR in cutting analysis: 1) VR technology provides a completely standardised and controlled laboratory environment [23], 2) VR has the potential to improve functional assessment [28], and 3) VR externalises focus to the realistic scenario, away from the injured joint [29]. This forces the body to rely on automatic motor control and improves automating movement patterns for RTS [28]. In summary, VR provides both experimental control and high-fidelity performance measurements, along with the potential to improve RTS rehabilitation programmes [23].

This study explores the feasibility of a VR-based assessment tool for evaluating cutting movements to enhance the ecological validity of sidestepping cutting analysis. The aim of this paper was to detail the design, development, and pilot of the VR application. The objectives were to: 1) identify the essential spatiotemporal demands of cutting tasks and how these can be manipulated, 2) create a VR environment and avatar with parameters that can be changed to alter those spatiotemporal demands, 3) optimise the system and determine the cutting parameters to remain constant throughout a study, 4) pilot the system and present the data on a single subject level.

The objective of the pilot case study was to identify the biomechanical fidelity of the VR environment and the effects of these cutting stimuli by analysing knee kinematics, kinetics, and personal perceptions of VR application fidelity. It was hypothesised that: 1) in the physical world and a realistic VR emulation of the physical world with arrows utilised as a directional cue, there would be no difference between conditions in knee abduction angles and moments, pelvis or foot velocity, or perceived realism, emotional responses, and perceptual-cognitive demands of the cutting task; 2) in the realistic VR emulation of the physical world with an avatar opponent utilised as a directional cue, as opposed to arrows, there would be an increase in knee abduction angles and moments, an increase pelvis and foot velocity, and an increase perceived realism, emotional responses, and perceptual-cognitive demands of the cutting task.

## 2. Theoretical underpinnings

### 2.1. Anterior cruciate ligament injuries

The ACL of the knee is essential for valgus and rotational stability, preventing the knee from translating and rotating excessively and keeping the femur in opposition with the tibia [30,31]. Non-contact ACLI are multifactorial in nature, with intrinsic risk factors, including biomechanical, neuromuscular, and genetic contributors, and extrinsic risk factors, such as environmental variables, types of sports (in particular football), playing techniques (predominantly regarding cutting, landing and jumping), and training patterns [32–34]. Furthermore, it has been identified that females have a 2.2-fold higher incidence of ACLI [34].

Both physical and psychological implications on athletes and their performance can be extreme and long lasting, with a compromised capacity to return to sports and enhanced re-injury risks [35]. The long-term societal financial costs of ACL reconstruction (ACLR) may be as high as US$38,000 per individual [36].

The strategies for prevention are critical, as are methods to limit the severity of ACLI. Preventive strategies have included sport-specific prophylactic programmes, specialised strength and neuromuscular training (NMT), and biomechanical and physical assessments to address injury-prone positions [31,34,37,38]. More recently, the potential of emerging technologies, such as VR, has come to light in enhancing research and injury-prevention strategies [39]. Particularly, one that allows for a personalised and multifaceted approach to prevention is essential for addressing the various risk factors [34].

### 2.2. Existing VR applications and novel features proposed

The potential of a VR application in the analysis of cutting manoeuvres has been well established [25,26,28,40], see S1 Table. VR has been used to alter laboratory environments and cutting task presentation [25], with the capacity to measure training transfer to realistic sport performance [26]. When cutting to the directional cue of an arrow in environments true to real world, movements in VR are generally comparable to those outside of VR [40]; however, effects on kinetics are possibly subject specific [40]. Most recently, in a move towards integrating VR as a feasible rehabilitation tool, Van Wallendael et al. [28] presented at conference as being one of the first to use commercially available, immersive VR to cut in a realistic sporting scenario with an avatar. They investigated the value of non-immersive Extended Reality (XR) and immersive VR on movement during RTS assessment of male soccer players post-ACLR, compared to healthy control players. Van Wallendael et al. [28] concluded that VR has the potential for enhanced kinematic sensitivity in detecting movement deviations during RTS evaluations post-ACLR. The unique contribution of this recent work has been the realistic simulated interaction with the avatar: heading a ball kicked by a virtual teammate and cutting to perform a quick return pass. The overall trend has been to deliver increasingly realistic VR environments and increasingly interactive sporting scenarios, allowing athletes to anticipate or react to representative environmental variables in true-to-life simulations.

Despite the rapid progression in this field, there has yet to be a VR application designed for the assessment of cutting in sport that is quantifiably true to real-world environments, with scenarios dependent on athlete movement, and the ability to alter spatiotemporal demands of the cutting task. In a logical progression from the existing work, the current VR application offers an environment with highly realistic visuals to within a 2 cm accuracy of the physical world, with the approach of the avatar instigated by the athlete’s movement in the virtual environment, and the ability for a ‘non-programmer’ to alter the demands of the task trial-to-trial by manipulating the avatar’s cutting parameters with simple input. Table 1 highlights how the current VR application is informed by and differs from applications previously developed. The examples are not exhaustive but are representative of the transition in research in this field.

**Table 1:** Features of VR applications designed to assess cutting manoeuvers.

	Visual stimuli to trigger cutting to unanticipated direction	Fully immersive VR (use of headset)	Use of commercially available headset	Cutting around a 3D virtual avatar	Highly realistic virtual environment	Avatar movement instigated by headset user position	Alterable avatar cutting parameters
Cortes et al. [25]	✔						
Kiefer et al. [26]	✔	✔		✔			
Lei and Cheng. [40]	✔	✔	✔				
Van Wallendael et al. [28]	✔	✔	✔	✔			
Current VR application	✔	✔	✔	✔	**✔**	**✔**	**✔**

## 3. Methods and materials

The step-by-step protocol for the current study can be accessed at protocol.io (see S2–S5 Protocols): https://www.protocols.io/view/lab-protocol-for-a-realistic-virtual-reality-appli-q26g758wqlwz/v1 [Accessed: 11/06/2025].

(DOI: https://doi.org/10.17504/protocols.io.q26g758wqlwz/v1 [Accessed: 11/06/2025]).

The VR application has been made available for download from the following GitHub repository: https://github.com/HannahKTang/VR-for-movement-assessment-in-sport.git [Accessed: 11/06/2025].

(DOI: https://doi.org/10.5281/zenodo.15102390 [Accessed: 11/06/2025]).

### 3.1. VR application design and development

#### 3.1.1. Experimental set-up.

The present study and VR environment were based on a typical laboratory-based cutting assessment. There were three experimental conditions, each having a different form of stimulus acting as a directional cue: 1) physical world with arrows; 2) VR emulation of the physical world with VR arrows, 3) VR emulation of the physical world with an avatar opponent (Table 2).

**Table 2:** Cutting scenarios.

Cutting scenarios	Environment and task
Scenario 1 – Non-VR Arrows	In the physical world, three stimuli were presented on a TV screen (110 cm): a cross sign for stop, a left arrow for cut left, or a right arrow for cut right.
Scenario 2 – VR Arrows	In a VR emulation of the data collection area, three stimuli were presented on a direct VR replication of the physical TV screen: a cross sign for stop, a left arrow for cut left, or a right arrow for cut right.
Scenario 3 – VR Avatar	In the same virtual emulation of the biomechanics laboratory, individuals reacted to the blocking manoeuvre of a VR avatar.

The VR environment was based on the laboratory in which cutting takes place. The direct replication of the arrow condition in the VR environment acted as a control. The environment was designed with a high level of face and construct validity, in that it was designed to look and ‘feel’ real and an accurate representation of a cutting task [41]. However, a control condition is needed to determine if the simulation elicits realistic motor movements, or acts as a confounding variable [41]. Similar to the work of Lei and Cheng [40], the ‘VR Arrow’ condition determines the level of biomechanical fidelity of the VR environment [41]. It would also provide an environmentally identical setting to compare arrow to avatar stimuli. At this stage of application development, the VR application is only intended for use in the lab that is emulated, specifically to act as a control. In future work, other environments could be replicated using the same methods.

**Fig 1:**
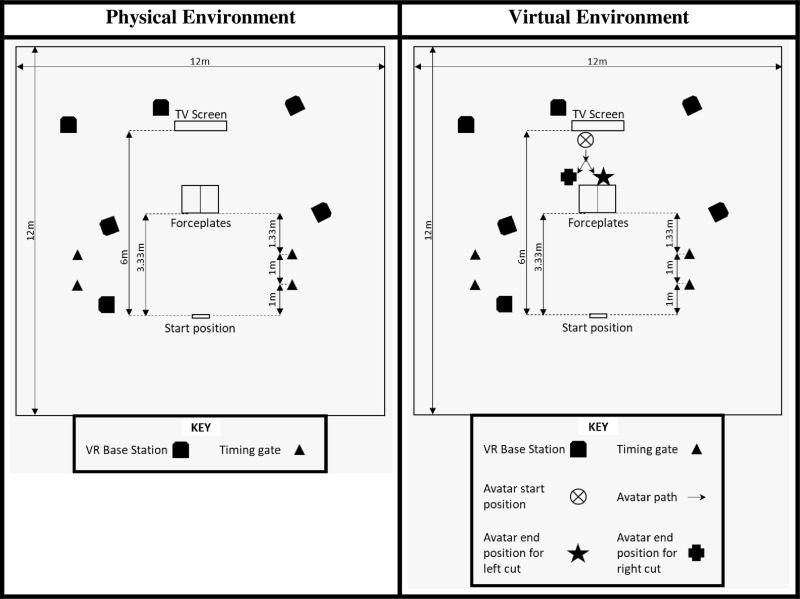
Experimental set-up in the physical environment and virtual environment.

The physical set-up was directly emulated in the virtual environment (Fig 1). This consisted of a 3.3 m run-up between the starting position and the near edge of two 60 x 90 cm force plates. Moving through the second timing gate triggered visual stimuli, either in the physical environment (arrows on a TV screen) or virtual environment (virtual arrows on a virtual TV screen, or avatar opponent approach). The participants could either react to the visual stimuli by stopping or cutting. When cutting, the cutting leg was planted on the left force plate directly in front of the TV screen or avatar. The avatar opponent was positioned to run directly towards the centre of the left force plate, and the avatar opponent then conducted a 30˚ cut to the left or right to direct the movement of the athlete with a blocking manoeuvre. There was 1.33 m from viewing the stimuli to reacting on the force plate. There was 6 m in either direction for follow-through steps. There was no trigger to visualisation delay.

The VR environment was delivered on an HTC Vive Pro system using the wireless adapter (HTC Corporation, Taiwan). This delivered a fully-immersive VR environment allowing for free ambulatory movement. Six base stations (2.0) tracked movement of the headset and were positioned for optimal coverage as seen in Fig 1.

#### 3.1.2. Features to alter cutting demands.

Control over variables allows the systematic delivery of visual stimuli to standardise the sporting scenarios and maintains internal validity of study findings [22]. It also meets the demands of researchers and clinicians with variable goals. When determining which variables should be modifiable in the VR application, there are many components to be considered which impact the mechanical capacity required to change direction successfully. The primary spatiotemporal demands can be met by the athlete’s navigation of: a) the direction in which they must turn [42], b) velocity of approach [43], and c) the angle of the cut [44]; see Dos’ Santos et al. [45], and Dos’ Santos et al. [46] for comprehensive overview. Table 3 describes the VR application input parameters and the expected movement outcome of the athlete as a result.

**Table 3:** Virtual manipulation of spatiotemporal demands of cutting task.

Spatiotemporal demand of cutting task on the athlete	Parameters altered by application input	Expected movement outcome of athlete
**Direction of cut: right/left**	• Direction of the avatar cut: right/left	The athlete cuts in the opposite direction to the avatar to evade the blocking move.
**Approach velocity**	• Position of timing gates: closer or further away from avatar. Movement through virtual timing gate triggers avatar approach. • Avatar running speed.	The athlete’s final approach velocity should increase when given a shorter distance or less time to react either due to: 1) a change in the position of the timing gates closer to the avatar or, 2) an increase in avatar speed.
**Angular degree of cut**	*N.B.* The input parameters for angle of cut were not manipulable by the end-user in the current application. A future application could incorporate the following features: • Degree of avatar’s cut • Start and end position of the avatar • Number of avatars • Position of teammates • Position of ball	Athlete changes angle of cut in response to play circumstances, including available space, the blocking position of opponent(s), movement of teammate(s), and the ball.

The application was designed so that the user could change the avatar’s parameters (i.e., avatar speed, avatar direction). Prior to each trial, there is a simple selection or input for each variable; these remain constant if left unaltered. For example, the specific approach speed of the avatar could be set at the average speed of the athlete, as recommended by Kiefer et al. [26]. While there is not yet an autonomous system in place to match the avatar’s movements to those of the headset user, the set-up can allow for the control of variables on a trial-to-trial basis.

Further to this, just as the user can adjust the physical environment, they can also adjust the virtual environment, and therefore how the athlete interacts with these tasks and the space around them. For example, the cue could be presented earlier or later by repositioning the VR timing gates relative to the VR screen, thus allowing the athlete more or less time to react; the athlete’s trajectory can also be controlled by changing their start position and direction by orientating the VR environment when it is overlaid onto the physical laboratory during calibration.

#### 3.1.3. VR development.

A 3D model replicating the laboratory where the pilot experiments would be conducted was created to scale. This included experimental equipment such as base stations, tripods, and timing gates. An infrared LiDAR sensor (Velodyne Puck lite (Velodyne Lidar, CA, USA)) was employed to capture the dimensions of the physical environment with a degree of accuracy of 2 cm. To recreate the laboratory, images of a colour palette (8bits RGB) were taken alongside 360° images of the laboratory using a GoPro MAX camera (GoPro, CA, USA) as a reference during the development process. The objective was to recreate the environment with a simplified scaled 3D mesh, using the free and open-source 3D computer graphics software tool Blender (Blender, Netherlands), while matching colours and textures to achieve a heightened sense of realism. Lighting was also taken into consideration during the 3D model creation process, and the resulting textures were baked to optimise the software performance when deployed in the Unity game engine, requiring less computing power as it achieves realism without the need for real-time lighting rendering. The virtual scene was then gamified using the game engine Unity (Unity Technologies, CA, USA).

#### 3.1.4. VR avatar body and movement.

In Unity game engine, avatar movements were rendered to allow the avatar to run towards the athlete and cut at 30° in a blocking manoeuvre. The avatar was positioned directly in front of the virtual TV screen, emulating a 1v1 scenario, common in training and RTS [47] and utilised by Cortes et al. [25] and Kiefer et al. [26]. To increase realism, the avatar swayed in their starting position until their movement was instigated. When the athlete moved through the second virtual timing gate, the avatar approached and conducted a 30˚ cut to direct the movement of the athlete with a blocking manoeuvre. A 30˚ cut is common in football [48] and ensured the avatar remained central in the field of view. The cut was identifiable but subtle enough to require continued attention and tax decision-making processes. As a result of the realistic avatar locomotion, the athlete was then able to respond by cutting in the opposite direction to evade them.

Certain compromises were made regarding the ecological validity of the VR application. At this stage of application development, experimental rigour was prioritised by reducing confounding issues and improving internal validity. This was prioritised to validate the application to a certain technology readiness level. The goal of the foundational work was to establish what effects a transition from arrow to avatar creates in controlled conditions, identifying its potential as an application before more ecologically valid alterations can be incorporated.

Realistic representations of bodies are an ongoing limitation in this field [26]. The avatar was formed of plain white segments as if wearing a metallic suit. It is widely accepted that individuals will respond to an avatar in different ways depending on their own demographics and features of the avatar (e.g., gender, race) [49,50]. It was unknown which participants would take part in the pilot. To prevent participant bias, and the participant reacting differently to the avatar because of their characteristic, a generic avatar was selected. However, this could detract from the sense of realism. In future VR applications, it could be possible for the user of the VR application to select an avatar with features of choice regarding gender, race, and disability status, such as those produced by CreatorCustom [51].

Alongside the limitations regarding aesthetic representation of the avatar’s body, there were also limitations relating to avatar movement. The virtual opponent has a fixed movement pattern and does not replicate the reaction pattern of the headset user. Therefore, the avatar does not reflect the dynamic uncertainty of a game situation to the same extent as an opponent with variable movement patterns. This may reduce the ability to conduct a fully naturalistic cut. Future work may look at different methods of rendering the avatar’s movements to improve ecological validity and transference to the real world, as this is an ongoing issue in this research field [27]. The method of animation could also incorporate honest and deceptive signals used to predict the movement of the opponent [52], i.e., an avatar that is able to ‘fake’ a cut.

The speed of the avatar may not accurately match that of the athlete. The current application attempts to address this in two ways: 1) the ability to alter avatar speed to match the average speed of the athlete, and 2) the feature that ensures the avatar cut is instigated by the close approach of the athlete. However, the approach speed of the athlete may vary between trials and within different phases of the approach. Avatar speed was constant all the way to the point of interception at the force platform area. For the case study participant, avatar speed was fixed at 2 m/s and they had approximately 0.66 s to react to the visual stimuli (comparable to reaction times seen in previous work of 0.5 s [57] to 0.7 s [58]). Their average approach speed measured via timing gates at 2.29 m/s. However, for the last 50 ms prior to pivot foot contact, the athlete’s speed would have reduced significantly compared to the preceding seconds during approach. In initial pilot work, this has not impacted the ability to conduct a naturalistic cut around the avatar; however, in future work, the avatar could potentially mirror the velocity of the headset and, the VR avatar opponent could have a decreasing curvilinear speed profile in the final approach steps. If the avatar were ‘yoked’ to the athlete as suggested by Kiefer et al. [26], this may remove the benefit of standardised stimuli. However, for this potential, pre-recorded 3D motion capture data to animate the avatar was avoided [26]. As the avatar movements are currently systematic and are the same between each trial, the implications for any biomechanical results need to be considered when drawing conclusions.

#### 3.1.5. Developed virtual environment.

**Fig 2:**
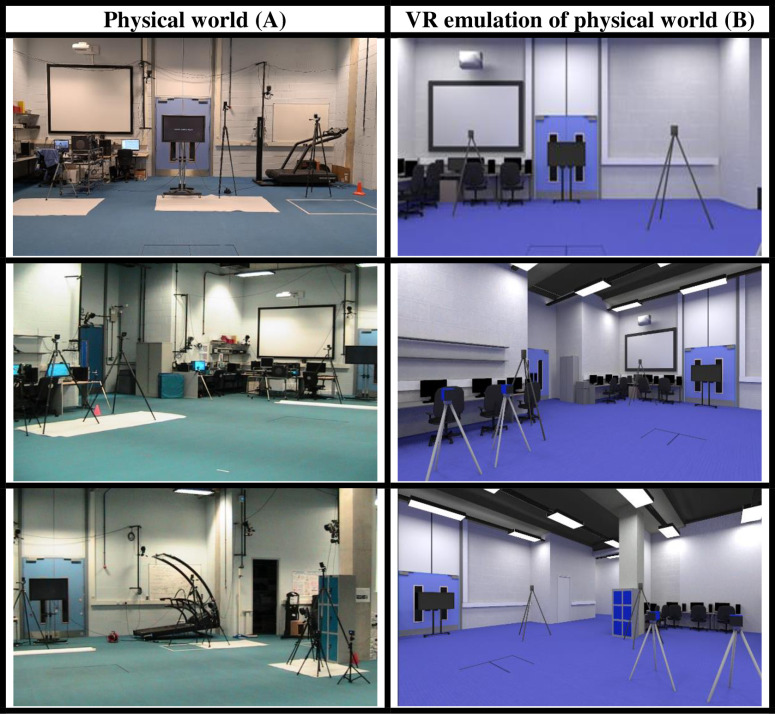
Replication of data collection laboratory (column A) as virtual reality environment (column B).

**Fig 3:**
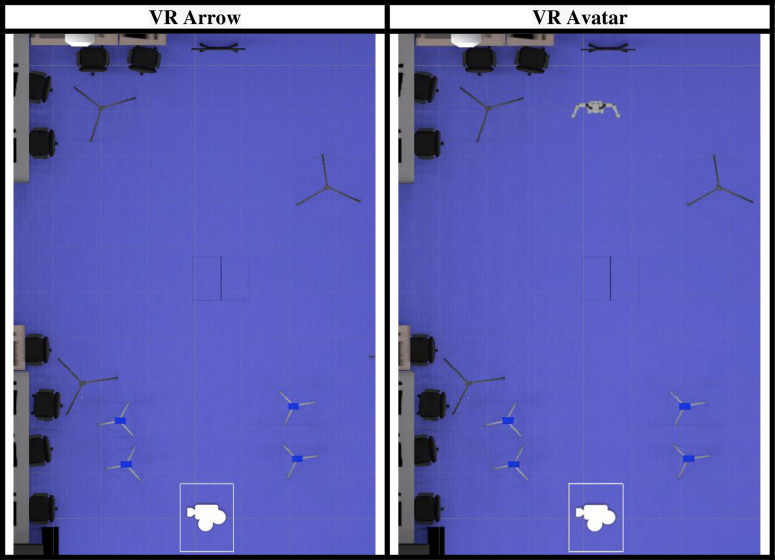
Experimental parameters in the virtual world as seen on the Unity game engine in a view from above, using arrows as visual stimuli and using avatar cutting scenario.

The physical environment and its emulation in the virtual environment, deployed in the Unity game engine, can be seen in Fig 2. Fig 3 shows the experimental parameters in the virtual world as seen on the Unity game engine in a view from above, with the TV screen and the addition of the avatar.

**Fig 4:**
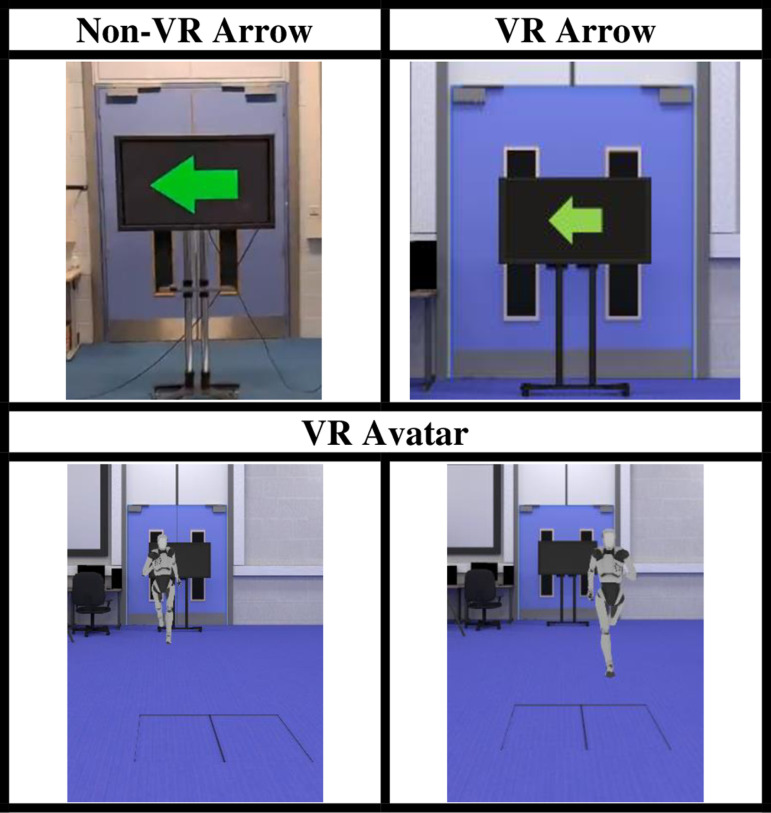
Visual stimuli displayed to inform athlete to cut left: arrow in the physical environment (‘Non-VR Arrow’), arrow in the virtual environment (‘VR Arrow’), and avatar in the virtual environment conducting blocking manoeuvre (‘VR Avatar’).

As a result of the design and development of the VR application, the following visual stimuli can be presented to an athlete indicating for them to perform a left cut (Fig 4). Only left cut data was processed in the current study.

#### 3.1.6. User interface in Unity game engine.

**Fig 5:**
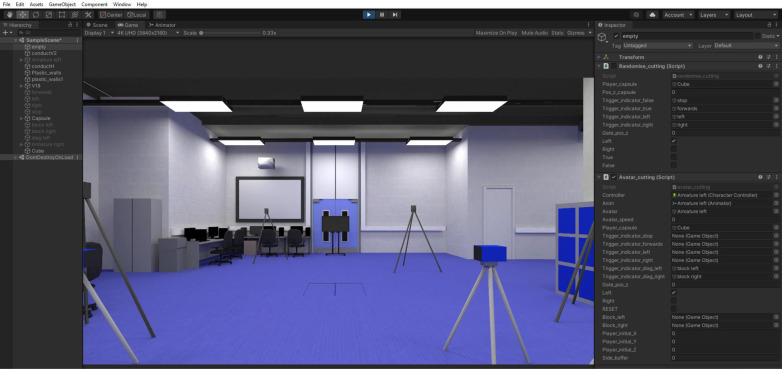
Image of game engine scene.

**Fig 6:**
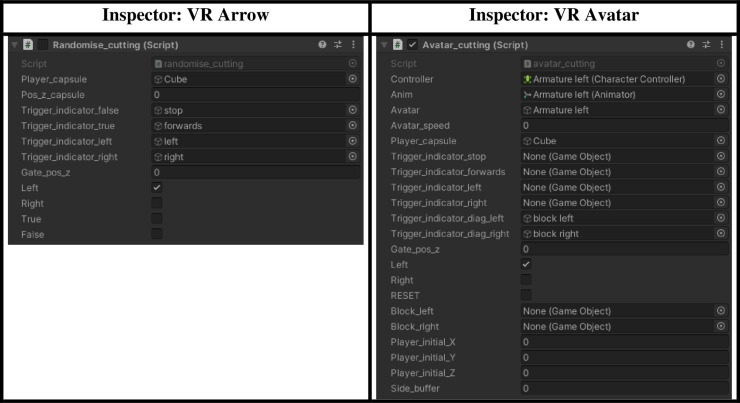
Inspector presenting options for user input for ‘VR Arrows’ and ‘VR Avatar’.

The VR application was in a development phase and alterations were consistently being made via the Unity game engine. Using the Unity Inspector, the VR application can be run by someone with no coding experience. However, further development would see the application shipped to a mobile device with a simpler user interface. See Fig 5 for image of the virtual scene displayed on the Unity game engine. To the left of the screen is the ‘Hierarchy’ that contains virtual objects to be selected as necessary. To the right of the screen is the ‘Inspector’, presenting options for user input. The Inspector (see Fig 6 for enlarged image) offers a simple interface to input numerical values of avatar speed and gate position, and to select direction of cut. Gate position is also alterable via click-and-drag.

### 3.2. Pilot methods

#### 3.2.1. Participants.

The VR application was piloted (Liverpool John Moores University Research Ethics Committee reference: 22/ENR/004). Four pilot participants provided written informed consent to take part in the study and to publish these case details, as outlined in the PLOS consent form. Piloting was conducted in accordance with the Declaration of Helsinki. The participants were medically screened, primarily ensuring no musculoskeletal complaints in 6 months, or issues with vision, or balance and neurological impairment.

In an ongoing process of system optimisation, the pilot process involved four participants, each attending the lab on separate occasions over the duration of one month. The piloting process involved two stages. The first stage involved three participants and focused on initial system testing and parameter optimisation. Designed with the intention of improving system development, this was then used after each session to improve the virtual scenarios. Having reached a functional system, the second stage focused on one individual in a case study analysing data output on a single participant level (female, 31 years old, 1.73 m, 72.3 kg, cutting novice, no previous VR use). This involved a full set of kinematic and kinetic data.

#### 3.2.2. Experimental protocol.

**Fig 7:**
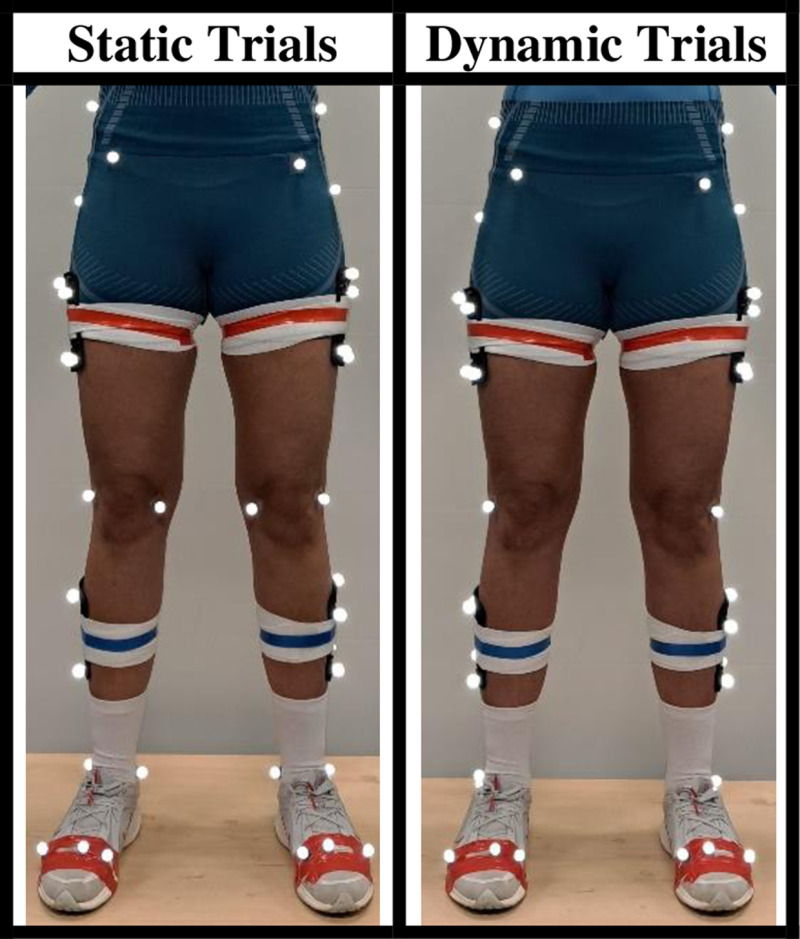
Marker model from front during static trials and dynamic trials with medial knee and ankle markers removed.

A marker model was applied. This consisted of 43 reflective markers placed on the pelvis and legs, including segment-defining markers and four-marker clusters on rigid plates used for dynamic tracking of the thighs and shanks. Medial knee and ankle markers were removed for data collection to allow for unhindered movement (Fig 7).

A Qualisys 14 infra-red camera motion capture system (Qualisys, Sweden; model: Arqus A12) recording at a sample rate of 250 Hz was used to capture the position of a marker model set. One Kistler force plate of 60 x 90 cm (Kistler Group, Switzerland; model: 9287C) collected kinetic data at a sampling rate of 1500 Hz.

Prior to each condition, the participant was provided with incremented familiarisation tasks repeated in four stages: 1) walk forward and stop at presentation of stimulus; 2) jog forward and stop at presentation of stimulus; 3) walk entire manoeuvre; 4) jog entire manoeuvre. This was repeated for a stop sign, left cut and right cut. Familiarisation time was not capped.

After task familiarisation, the pilot participants involved in system optimisation conducted 15 successful left cuts under the three conditions (5 ‘Non-VR Arrow’, 5 ‘VR Arrow’, 5 ‘VR Avatar’), and the case study participant performed a total of 25 successful left cuts under the three conditions (9 ‘Non-VR Arrow’, 9 ‘VR Arrow’, 7 ‘VR Avatar’). These were unanticipated, 90° cutting manoeuvres to the left (with interspersed right cuts and stops – 3:1 ratio of left:right or stop). Only left cuts were processed. Approach speed was self-selected to impose minimal intervention on the cut and for ethical considerations. The participant was told to ‘run as quickly as possible whilst still feeling safe’. They were also informed of the visual stimuli being presented and were actively told to ‘cut in the opposite direction to the avatar blocking manoeuvre’.

After the motion trials, the participant answered an eight-question questionnaire (Table 4). This determined any impacts of: dominant leg use, prior participation in biomechanical research or sport, prior VR use. The questionnaire also identified participant perspectives regarding whether the VR simulation captured fundamental features of the physical environment and a real opponent cutting scenario, as well as the participant’s opinion on the VR application eliciting realistic behaviours [41]. The questions posed to the participants directly related to measurements of presence and task engagement to determine, within each condition, the level of: 1) physical fidelity – realism provided by the VR application, 2) emotional fidelity – emotional response to the stimuli, and 3) perceived psychological fidelity – replication of perceptual-cognitive demands of the cutting task [41].

**Table 4:** Eight-question questionnaire.

1. Which is your dominant leg?
2. Have you participated in a study that has measured quick directional change (cutting) trials before?
3. Have you ever competed in a sport (at any level) where you would do a movement like this? If yes, which and at what level?
4. How many hours would you normally spend using a VR headset in any given week?
5. Did you feel like the virtual environment (virtual lab room) was similar and different from the real environment? If yes, how?
6. Do you feel like you moved differently in the virtual environment compared to the real environment? If yes, how?
7. Did you feel like there was a difference between how the arrows directed your movement compared to the avatar? If yes, how?
8. Is there anything else you feel would improve the VR experience either as a user, or for sports, or for rehabilitation?

#### 3.2.3. Data processing.

**Fig 8:**
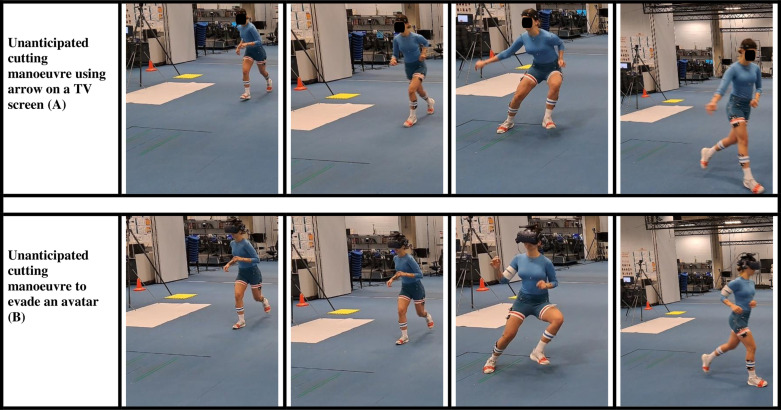
Participant cutting without a headset (row A) and with a headset (row B).

***Initial system testing and parameter optimisation*:** The initial stages of testing relied on a Likert-scale-based ‘System Usability Scale’ (S6 Table) and questionnaire (Table 4) specifically designed to gain personal feedback and input from the first three participants and aid discussion of system issues. It was not delivered to compare qualitative input between participants or substantiate the case study pilot, which was stand-alone. Throughout the session, the researcher also created notes on user and system-related events. After each piloting session, these were used to enhance the functionality and usability of the system. It also determined the optimal parameters for a naturalistic cut that would remain constant for systematic delivery of the task from trial-to-trial. This was finalised prior to the attendance of the next participant. See Fig 8 for image of participant cutting in and out of VR.

***Pilot case study: Spatiotemporal measures, k***inematics and kinetics****:** Markers were labelled, raw data was gap filled, and files were exported in.c3d format to a skeletal modelling software Visual3D (C-Motion, ON, Canada). The raw kinematic data was filtered at 20 Hz; kinetic data was then match filtered [53]. The relevant skeleton segments of the pelvis and right leg were modelled. For comparison to data collected in existing studies, all knee moments were normalised by the product of the participant’s mass (72.3 kg) and height (1.73 m). Gait events were applied, specifying ground contact by identifying the ‘right heel strike’ when the heel makes contact with the ground and the ‘right toe off’ when the foot no longer makes contact with the ground. Model-based data was computed for joint angles and internal net moments of the knee. Centre of gravity velocities were calculated for the pelvis and right foot. Velocity was calculated from 50 ms prior to Initial Contact (IC) (i.e., 50 ms prior to 10 N detected by the force plate) to the instant of IC.

***Questionnaire*:** The volume of questionnaire data was not comparable to that of a study requiring in-depth qualitative analysis. All responses were presented in the results section in the same form as they were collected.

#### 3.2.4. Data analysis.

***Pilot case study: ***Spatiotemporal measures, k***inematics and kinetics*******:** Mean knee frontal plane angle and moment values across trials for each of the three visual conditions were all normalised to the stance phase (heel strike to toe off) of the cutting step. The mean average curve was determined and plotted, with corresponding standard deviation (SD). Adduction was reported as positive.

Mean curve and SD were also plotted for centre of gravity (COG) velocity component in the forward direction of movement leading into the turn for: 1) the pelvis, as an indicator of centre of mass (COM) velocity of the whole body, and 2) right foot (cutting foot).

To accurately describe the severity of the turning angle, the angle of cut for each condition was calculated as the maximum angular change between the vector of the pelvis COM at approach and exit of the cut stride [54]; i.e., between the swing phase of the pivot leg entering and exiting the cut. Angle of cut was tabulated for each condition.

As the current work is an initial case study of one participant, a mean curve was plotted and SD acted as an indicator of variability in the participant’s performance. Using SD as a quantification of signal from noise allowed relative comparison between conditions, i.e., no overlap of SD being an indicator of real difference between conditions. Further to this, a table presented values for each outcome variable (kinematics and kinetics during the stance phase and velocities in the 50 ms prior to IC). The table reported mean average value, SD, and 95% confidence interval (CI), alongside the mean of the peak values (reported as maximum absolute values with corresponding SD). A further table presents the mean angle of cut for each condition.

## 4. Results

### 4.1. System optimisation results

Issues presented during system optimisation are listed in S7 Table. These included any topics brought up in discussion or noted as user- and system-related events, issues raised in the questionnaire, or items listed as 2 or below in the System Usability Scale. For an overview of action taken for system optimisation, see S7 Table.

### 4.2. Case study results

#### 4.2.1. Spatiotemporal measures.

**Fig 9:**
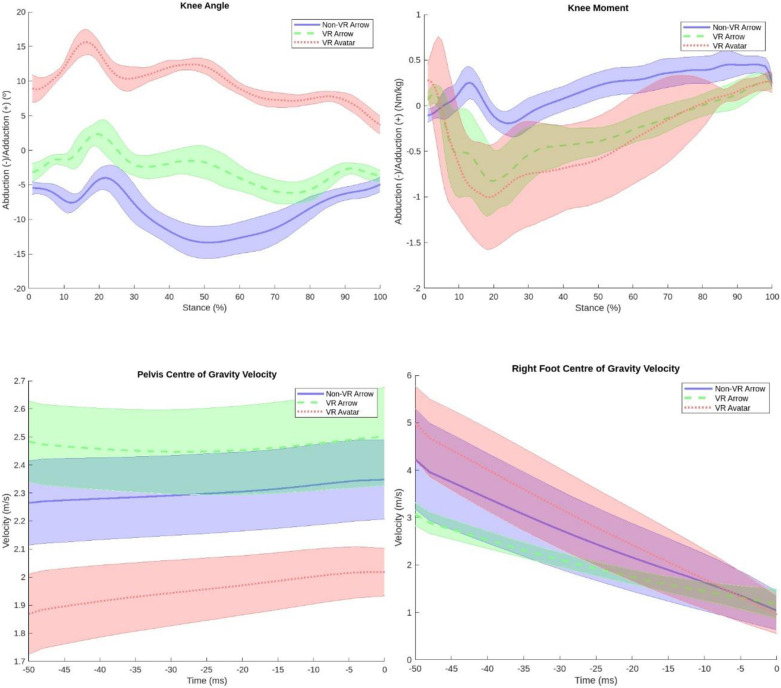
Pelvis and right foot centre of gravity forward velocity component 50 ms prior to Initial Contact (0 = Initial Contact) (A); knee abduction-adduction angle (B); knee abduction-adduction moment (C).

***Pelvis velocity*:** Regarding pelvis velocity (Fig 9), there was substantial overlap in SD between the ‘Non-VR Arrow’ and ‘VR Arrow’ condition. The mean pelvis COG velocity in the 50 ms prior to IC was greatest in the ‘VR Arrow’ condition (2.5 m/s ± 0.2), followed by the ‘Non-VR Arrow’ condition (2.3 m/s ± 0.1). There was no overlap in SD with the Pelvis COG in the ‘VR Avatar’ condition, which was markedly lower (2.0 m/s ± 0.1). The spread in the pelvis velocity was similar in each condition.

***Right foot velocity*:** Mean right foot COG velocity in the 50 ms prior to IC (Fig 9) was greatest in the ‘VR Avatar’ condition (2.9 m/s ± 0.7), with a lot of overlap in SD with the ‘Non-VR Arrows’ condition (2.0 m/s ± 0.8). The right foot COG velocity was particularly lower in the ‘VR Arrows’ condition (2.0 m/s ± 0.1), as was the spread of data in this condition.

Peak and mean values for all graphed data can be seen in Table 5

**Table 5:** Kinematic, kinetic and spatiotemporal measures.

	Condition	Peak	±SD	Mean	±SD
**1**	**Knee Angles (**°)			**(Abduction (-), Adduction (+))**	
	Non-VR Arrow	13.6	2.4	−8.8	1.4
	VR Arrow	6.5	1.4	−2.7	0.9
	VR Avatar	15.7	1.8	9.8	0.4
**2**	**Knee Moment (N/kg)**			**(Abduction (-), Adduction (+))**	
	Non-VR Arrow	0.5	0.1	0.2	0.1
	VR Arrow	0.9	0.4	−0.3	0.2
	VR Avatar	1.1	0.5	−0.4	0.4
	**Velocity (m/s) of segment centre of gravity in the 50 ms prior to heel strike**				
**3**	**Pelvis**				
	Non-VR Arrow			2.3	0.1
	VR Arrow			2.5	0.2
	VR Avatar			2.0	0.1
**4**	**Right foot**				
	Non-VR Arrow			2.6	0.8
	VR Arrow			2.0	0.1
	VR Avatar			2.9	0.7

***Angle of cut*:** See Table 6 for angle of cuts. Angles of cuts for all trials averaged at 69.6° with a variation of 12.4° between the mean values for each condition. The average cut for the ‘Non-VR Arrows’ and ‘VR Avatar’ condition only differed by 1.1°. The ‘VR Arrows’ condition presented a much lower angle of cut and the greatest variation 61.7 ± 6.4.

**Table 6:** Mean angle of cut entering and exiting cut step.

Condition	Angle (°)	
	Mean	±SD
Non-VR Arrow	73.0	3.5
VR Arrow	61.7	6.4
VR Avatar	74.1	4.3

#### 4.2.2. Kinematics.

Distinct difference in mean knee angle existed between each condition (Fig 9). The mean knee angle in the ‘Non-VR Arrow’ and ‘VR Arrow’ conditions presented abduction values (−8.8° ± 1.4 [CI: −9.5–8.2]; −2.7° ± 0.9 [−3.2–2.2], respectively). However, the mean knee angle during the ‘VR Avatar’ condition presented adduction values (9.8° ± 0.4 [9.2 10.4]), with lower variability between trials. Only the ‘VR Avatar’ condition had the average curve clearly in the positive adduction region. For the VR conditions, mid-stance (often associated with the point of turning) indicated slight adduction, as suggested by visual inspection of the plots.

#### 4.2.3. Kinetics.

There was overlap in SD between each condition for mean knee moment (Fig 9). However, this overlap was greater between the VR conditions. The mean knee moment during the ‘VR Arrow’ condition and the ‘VR Avatar’ condition presented abduction values (−0.3 N/kg ± 0.2 [−0.3–0.2]; −0.4 N/kg ± 0.4 [−0.5–0.3] respectively). Conversely, the mean knee moment in the ‘Non-VR Arrow’ condition presented adduction values (0.2 N/kg ± 0.1 [0.2 0.2]). The VR conditions had greater variability between trials, with the greatest variability in the ‘VR Avatar’ condition. The VR conditions displayed a higher and earlier peak at IC, highest and earliest in the ‘VR Avatar’ condition, as identified on visual inspection of the plot.

#### 4.2.4. Questionnaire.

The questionnaire responses from the case study participant are presented in Table 7 in the same form as they were collected. These indicated the participant’s perception on the VR environment’s: 1) physical fidelity, e.g., ‘lab looked similar’, 2) emotional fidelity, e.g., ‘I was more cautious’, and 3) perceived psychological fidelity, e.g., ‘difficult to evaluate the direction [the avatar opponent] would turn’.

**Table 7:** Questionnaire data.

**Did you feel like the virtual environment (virtual lab room) was similar and different from the real environment? If yes, how?** The overall lab looked similar (shapes, layout) but differences existed in location of certain objects. There was a ‘zoom-in’ sensation when approaching the avatar.
**Do you feel like you moved differently in the virtual environment compared to the real environment? If yes, how?** I was more cautious in VR. I stopped at any sound and sooner after cutting.
**Did you feel like there was a difference between how the arrows directed your movement compared to the avatar? If yes, how?** Arrows were easy to process. The avatar was more difficult to process as it seemed to be running in a straight line at the beginning and was difficult to evaluate the direction it would turn, but it was also closer to a real situation. The zoom-in effect made it more difficult.
**Is there anything else you feel would improve the VR experience either as a user, or for sports, or for rehabilitation?** If copying the real world, the objects will always have to be in the same position as the real world. I don’t know how an injured patient would respond to the environment.

## 5. Discussion

This design and development study utilised a VR application to assess cutting movements in environments either with or without an avatar opponent cutting scenario. To the authors’ knowledge, this is the first VR application dedicated to this purpose with a highly realistic VR environment, VR scenarios instigated by athlete movement, and spatiotemporal demands of the cutting task that can be manipulated by altering avatar movements. Arguably, in these preliminary findings, the data indicated a strong level of conditions effect based on differences identified in knee kinematics and kinetics during ground contact, as well as approach velocities averaged over the 50 ms leading into IC.

As hypothesised, there was a similarity between conditions using directional arrows, both physical and VR, regarding knee kinematics and pelvis centre of gravity velocity. The mean knee angle in the ‘Non-VR Arrow’ and ‘VR Arrow’ control conditions presented abduction values and the ‘VR Avatar’ condition adduction values, suggesting the realistic VR emulation of the physical world does not act as a confounding variable. The VR-emulated environment may therefore prove sufficient as a control. The similarity of movement in the arrow conditions, Non-VR and VR, also implies suitable motor learning could occur in the VR environment and successful transfer of skill training may be possible [41,55].

Contrary to the hypotheses, when cutting around the VR avatar opponent, compared to when cutting around Non-VR and VR directional arrows, there were distinctly greater knee adduction angles. Knee adduction angles may not be associated with ACLI risk [56]. However, this is comparable to a study by Schroeder et al. [57] where cutting around a live human opponent, as opposed to an arrow, also resulted in greater knee adduction in male participants. This suggests that moving around an avatar opponent imposes the same spatiotemporal demands as cutting around a live opponent. Furthermore, in the current study, there was also a distinctly slower mean pelvis COG velocity when cutting around the avatar opponent (0.5 m/s slower than ‘VR Arrows’), but a slightly greater right foot velocity when entering the cutting step (0.9 m/s faster than ‘VR Arrows’). Participants were able to maintain the angle of cut in each condition, and this would not account for the results. This suggests that the introduction of the VR avatar opponent slowed the participant’s approach and altered knee frontal kinematics and spatiotemporal measures. Within the scope of this study data, attributing a cause may be premature.

Speculative interpretation of the findings indicates that when faced with unanticipated cutting scenarios, the participant adopted a different and more cautious movement strategy. The participant adapted with an increasingly cautious approach when processing more complex information, as seen with the approach to the avatar opponent. Based on the feedback from the participant (‘The avatar was more difficult to process’, ‘closer to a real situation’), it is possible that, even with familiarisation in each condition, the interaction with the avatar opponent introduced a more unpredictable scenario with realistic and complex decision-making processes and responses. This may have caused a reduction in approach velocity to gain processing time. As found by Cortes et al. [25], by increasing the number of choices during the unanticipated condition, participants had to slow down to closely observe the screen to properly react and execute the correct task. It is also in keeping with the more recent work of Lee et al. [58], who identified that perception of game-realistic visual information, i.e., using defender scenarios instead of arrows, contributed to a safer neuromuscular strategy. One could suggest that there is a sliding scale of progressively increasing realism: 1) anticipated cutting of a known cut direction, 2) unanticipated cutting with directional arrows, and 3) an avatar opponent adding a new element of unanticipated and ‘unpredictable’ cutting. The closer the avatar emulates human movement, the more unpredictable.

To further support the argument of avatar unpredictability, adapting a cutting strategy when approaching the avatar opponent, to interpret and react to its change in movement, may have reduced awareness of the precise timing of foot-ground impact. Supporting the hypotheses, the velocity of the stepping foot was greatest when moving into the pivot to avoid the avatar opponent. This indicates that collision at ground contact may have occurred prior than expected. As with adapting to the first step of a new running surface [59], this dual-task nature of processing avatar-opponent interaction and coordinating movement may have implications for cutting leg stiffness. Stiffness levels may have been less prepared on ground contact than would normally be seen during more predictable cutting scenarios around arrows, i.e., navigating around the avatar prevented optimal stiffness [60]. The participant appeared to be caught unaware when approaching the avatar opponent. Familiarisation to the avatar contained more impact-hop pivot steps. During data collection, the participant would look at the avatar opponent repeatedly and for longer, and there appeared to be greater variability in the ground reaction force vector during weight acceptance. The culmination of these factors potentially increases the risk of injury from either impact loading or excessive motion. In this way, the VR avatar opponent may better capture the complexity of ecologically representative cutting tasks.

With regards to moment values, the introduction of the VR headset alone may have acted as a confounding variable. Although each condition displayed similar knee moment patterns, wearing the headset caused greater mean abduction moments, with earlier initial adduction peaks, potentially indicating more rapid and greater loading, and subsequently, an increased injury risk [61]. As identified by Lei and Cheng [40], more investigation is required to determine if the VR headset and/or system has a confounding effect on knee moment for specific individuals.

## 6. Limits of the current VR application and future directions

The current study presented case study piloting data and as such is not intended to be generalisable to greater populations. This limited the data that could be investigated and statistical analyses that could be conducted. A further proof-of-concept study with a greater number of participants could better determine if this application has the potential to improve authenticity of decision-making and movement. Furthermore, non-contact ACLI occurs during complex multi-planar movements [5,62]. Future work could also analyse movement about the transverse and sagittal plane. To better understand the forces on the knee during cutting, a biomechanical model of the knee can be applied, leading to dynamic adjustments of cutting strategies and landing techniques to reduce the risk of ACLI [63].

Realistic representation of the application user’s body is an ongoing limitation [40]. Virtual body segments of the athlete were not present in the virtual environment. Consequently, the athlete would not see their body when in VR. This could be added at a later stage.

Regarding study design, familiarisation was not capped for safety reasons. The participant may have been more familiar with one condition than another. However, familiarisation was incremented for each condition and this ensured a baseline understanding of each condition. Additionally, data from a single participant is used for the demonstration of the VR application using a relevant set of conditions to compare real-world (arrows on a screen) against a VR environment that mimics that real-world environment (as a control), and then a more realistic avatar in the VR environment. Future work could add a new dimension to this work by addressing the realism of the cutting response when compared to game situations.

A primary limitation is that approach speed was not controlled, as was the case in similar studies [25,26,40]. Allowing self-selected approach speed provided an understanding of the effects of visual cues on speed as an outcome measure; it also enhanced ecological validity. In practice, self-selected speed could provide information as to cutting strategy [64] and determine changes in performance [16]. Fundamentally, with the exploratory nature of the work and the potential safety considerations regarding confined areas and restricted vision, it would have been unethical to impose a minimum approach speed. Unlike the participants of Kiefer et al. [26], there was consistent motivation and a minimum speed was not required. However, variable approach speed within and between conditions does make the results susceptible to participant learning and fatigue effects. Fundamentally, results could be attributed to speed variation, which is the primary confounding variable of the study.

The pilot identified that the VR environment and headset introduced certain confounding variables, potentially affecting kinetic measurements in particular. As identified by the participant, ‘differences existed in location of certain objects. If copying the real world, the objects will always have to be in the same position as the real world.’ Additionally, ‘there was a ‘zoom-in’ sensation when approaching the avatar’. There was not a close proximity to objects, however, the potential for collision may always elevate anxiety. This is particularly the case when running at speed in a virtual environment with a potentially reduced visualisation and image quality under motion Zhao et al. [65] and reduced ability to investigate sounds, as commented on by the participant, ‘I was more cautious in VR. I stopped at any sound and sooner after cutting’. Furthermore, the headset measured at approximately 1 kg in total, including adapter and wires. This has been seen to cause some discomfort during dynamic actions, although it did not seem to cause dizziness as seen in similar studies using the same brand of headset [40]. This validates the need for the arrow control to ensure the headset is not affecting outcome measures.

The use of force plates in a future proof-of-concept work would not be recommended due to time constraints, force plate targeting effects, and due to the confounding impact of headset use on the kinetic results.

## 7. Conclusions

The current study presented the first known realistic VR application that is easy to implement for the analysis of quick directional change in sport. The novel features are a highly realistic VR environment, VR scenarios instigated by athlete movement, and spatiotemporal demands of the cutting task that can be easily altered by manipulating an opponent avatar. These preliminary results suggest that the VR-emulated environment may prove sufficient as a control and that moving around an avatar opponent did alter approach strategy. It did question the confounding impact of headset use, particularly on kinetics. However, this was a proof-of-concept piloting case study of one participant, whose performance solely acted as an indicator of variability between conditions. Future research could contain a larger sample size to detect statistical effects of differences for more definitive conclusions. This would also allow for the further assessment of issues raised, such as the ‘zoom in’ effect described by the participant. The VR application also has the potential to be shipped to a mobile device at a later stage of application development. With the realistic environment, and the avatar-based cutting scenario, this application has the potential to improve the ecological validity of cutting assessment and makes a VR application more accessible to researchers and clinicians.

## Supporting information

S1 Table Existing VR applications to assess cutting. (PDF)

S2 Protocol Lab protocol 1. (PDF)

S3 Protocol Lab protocol 2. (PDF)

S4 Protocol Lab protocol 3. (PDF)

S5 Protocol Lab protocol 4. (PDF)

S6 Table System usability scale. (PDF)

S7 Table Issues raised and solutions taken during system optimisation. (PDF)
