# Supplementary material for: Design, development and pilot of a realistic virtual reality application to analyse quick directional change in sport: Avatar cutting scenario with alterable parameters
Source: PLoS One. 2025 Jun 24;20(6):e0324941. doi: 10.1371/journal.pone.0324941 (PMC12186900; doi:10.1371/journal.pone.0324941)
Supplement: S1 Table — (PDF) [file pone.0324941.s001.pdf]

S1 Table. Existing VR applications to assess cutting

Authors	Research purpose	Participants	Intervention	Results	Conclusions
**Cortes, Blount (25)**	Assessed unanticipated and anticipated lower extremity biomechanics during side-step cutting using non-immersive visualization software.	13 female collegiate football athletes.	Opponent images delivered on large screen. Cutting contra-laterally to dominant foot, with angle between 35º to 55º.<br>5 successful sidestep trials amidst running stop or sidestep cuts.	Performing the unanticipated condition increased knee abduction angle (unanticipated: -7.2º ± 5.38; anticipated: -4.0º ± 5.38), and knee internal rotation (unanticipated: 8.1º ± 4.78; anticipated: 5.2º ± 6.58), and decreased peak knee internal adduction moment (unanticipated: 0.37 ± 0.36 Nm/kgm; anticipated condition: 0.52 ± 0.4 Nm/kgm).	ACLI studies should account for laboratory environment and task presentation.
**Kiefer, DiCesare (26)**	Developed fully-immersive VR cutting application to examine skill transfer after six weeks of neuromuscular training.	5 female varsity high school football athletes, compared to 2 controls (after data reduction).	On custom built headset, VR cutting scenario consisted of 1v1 defensive cut to remain in front of a VR avatar.<br>Pre- and post-training, 2 cutting trials left or right.	From pre- to post-training, athletes exhibited a non-significant 19% reduction in knee abduction (values solely plotted), and a significant reduction in internal hip rotation.	Sport-specific VR may measure training transfer to realistic sport performance, while allowing experimental control and high-fidelity performance measurements.
**Lei and Cheng (40)**	Ascertained biomechanical fidelity of lab-based VR environment using arrow-directed cutting.<br>Compared virtual and physical world both in anticipated and unanticipated cutting.	12 first-division collegiate male football players.	HTC Vive headset delivered a virtual lab environment, emulating the visual cue of a directional arrow. Cutting at 45º.<br>20 successful sidestep trials per participant amidst sidestepping or crossover.	Performance and knee biomechanical parameters were comparable in the lab and VR-emulated lab. Anticipation caused similar changes in velocity and knee kinematics/kinetics in both environments (bar larger peak flexion angle in VR). Changes in moment values between environments during phases of the cut were subject specific.	Movements between the lab and the VR emulation of the lab were generally comparable. VR effects on moments may be subject specific.

**Van Wallendael, Artois (28)**	Compared cutting and vertical jumping in typical laboratory conditions to the proposed VR (and XR conditions).	VR participants: male participants. 14 with history of ACL reconstruction, 17 matched controls.	Non-VR: Cutting or jumping in direction of a Lummic reaction light, cutting left or right, or jumping centrally.<br>VR (and XR): 1) virtual teammate kicks the ball towards the region above the user's head and user heads the ball, and 2) user cuts to perform a quick return pass, left or right.	VR-results revealed significantly greater knee flexion (average knee flexion ROM Non-VR: 46.63° ± 6.375; VR: 49.47° ± 6.44) and significantly greater abduction range compared to non-VR<br>(average knee abduction ROM Non-VR: 11.55° ± 7.52; VR: 14.33° ± 7.84. Maximum knee abduction Non-VR: 16.21° ± 11.07); VR: 19.73° ± 10.46).	VR-based kinematic analysis may improve sensitivity in detecting movement deviations during RTS evaluations post-ACLR. Therefore, XR and VR applications have potential when identifying ACLR-related deficits.
