# Supplementary material for: Design, development and pilot of a realistic virtual reality application to analyse quick directional change in sport: Avatar cutting scenario with alterable parameters
Source: PLoS One. 2025 Jun 24;20(6):e0324941. doi: 10.1371/journal.pone.0324941 (PMC12186900; doi:10.1371/journal.pone.0324941)
Supplement: S2 Protocol — (PDF) [file pone.0324941.s002.pdf]

Lab protocol for a realistic virtual reality application to analyse quick directional change in sport: Avatar cutting scenario with alterable parameters

Hannah K.M. Tang[1,2], Mark J. Lake[3,2], Richard J. Foster[3,2], Frederic A. Bezombes[1,2]

[1]School of Engineering, LJMU, UK;
[2]Current address: Liverpool John Moores University, Byrom St, Liverpool, L3 3AF;
[3]Research Institute for Sport and Exercise Sciences, LJMU, UK

Hannah Tang
Liverpool John Moores University

Collection Info: Hannah K.M. Tang, Mark J. Lake, Richard J. Foster, Frederic A. Bezombes . Lab protocol for a realistic virtual reality application to analyse quick directional change in sport: Avatar cutting scenario with alterable parameters. **protocols.io** **https://protocols.io/view/lab-protocol-for-a-realistic-virtual-reality-appli-d7rp9m5n**

Created: April 07, 2025

Last Modified: April 10, 2025

Collection Integer ID: 126479

Keywords: Virtual reality, Avatar, Arrows, Cutting, Quick directional change, Sport, Biomechanics

Funders Acknowledgements:
Liverpool John Moores University in the form of Doctor of Philosophy funding
Grant ID: Student ID 854667

Abstract

Quick directional change in sport (cutting) has high potential for injury. Cutting mechanics can be analysed to inform injury risk and return to sport. However, during research and rehabilitation, laboratory and clinical settings can make it difficult to assess cutting as these environments are not representative of real sporting scenarios. As a solution, virtual reality (VR) has been used to replicate sporting scenarios in a realistic way that can be standardised. The use of VR to improve the ecological validity of cutting analysis has been evolving for a number of years. The current research group designed and developed a VR environment that emulates the physical world and allows unanticipated-cutting manoeuvres to be analysed with: 1) VR arrows as a cue for cutting direction, and 2) a VR avatar-opponent blocking manoeuvre as a cue for cutting direction. The VR environment is highly realistic, avatar approach is instigated by the movement of the headset user, and simple input alters the avatar's movements and spatiotemporal demands of the cutting task. When implemented, this lab protocol assesses the effects of the different visual cutting cues on cutting mechanics. The lab protocol highlights how the testing protocol methodology is novel and addresses difficulties that have previously arisen when integrating VR technology with biomechanical equipment in cutting assessment. It explains how to access and implement the VR application alongside a motion capture system, as well as how to account for safety and feasibility when the participant's vision of the physical world is restricted. It also provides a protocol that can be used when collecting kinematic and kinetic data with participants. The step-by-step protocol provides an outline on how to implement the study proposed, and given the growing interest in improving ecological validity in laboratory-based biomechanical assessments, it can be used as reference in future research.

Safety warnings

Prior to starting the study, all individuals were informed of the safety provisions. This included:

- The role of the periphery foam on the floor.
- That the primary researcher would monitor movement.
- Individuals had a minimum of one additional visual 'spotter' (other than the primary researcher) who watched the participant when moving with a headset on.
- Participants were told to emergency stop if the command 'Stop!' was yelled at any time.
- Participants were to jog or run every recorded trial at their own 'safe maximum' (the maximum speed at which they felt safe). They were asked to continually monitor this. However, individuals were prompted to slow down if their speed became a safety risk.
- Individuals rested for a minimum of 3 minutes every 8 trials. A resting seat, outside of the capture volume, was identified for their use and they were told to request a break, if needed, at any time.

Ethics statement

Liverpool John Moores University Research Ethics Committee reference: 22/ENR/004.
Participants provided written informed consent to take part in the study and to publish these case details. The research was conducted in accordance with the Declaration of Helsinki. The participants were medically screened, primarily ensuring no musculoskeletal complaints in 6 months, or issues with vision, or balance and neurological impairment.

Before start

The VR application can be downloaded from GitHub, in-line with the licence outlined on GitHub:
https://github.com/HannahKTang/VR-for-movement-assessment-in-sport.git
The VR application can be referenced with the following DOI:
https://doi.org/10.5281/zenodo.15102390

Files

SEARCH

Protocol

NAME

Systems and setup

VERSION D7RK9M4W

CREATED BY

Hannah Tang
Liverpool John Moores University

OPEN →

Protocol

NAME

Data collection protocol: Screening and motion trial preparation

VERSION D7RM9M46

CREATED BY

Hannah Tang
Liverpool John Moores University

OPEN →

Protocol

NAME

Data collection protocol: Motion trials

VERSION D7RN9M5E

CREATED BY

Hannah Tang
Liverpool John Moores University

OPEN →

Protocol references

1. Bach M. The Freiburg Visual Acuity Test-automatic measurement of visual acuity. Optometry and vision science. 1996;73(1):49-53.
2. Bach M. The Freiburg Visual Acuity Test-variability unchanged by post-hoc re-analysis. Graefe's Archive for
Clinical and Experimental Ophthalmology. 2006;245:965-71. doi: https://doi.org/10.1007/s00417-006-0474-4.
3. Parede TRR, Torricelli AAM, Mukai A, Vieira Netto M, Bechara SJ. Quality of vision in refractive and cataract
surgery, indirect measurers. Arquivos Brasileiros de Oftalmologia. 2013;76:386-90. doi: https://doi.org/10.1590/S0004-27492013000600016.
4. Vancleef K, Read JC, Herbert W, Goodship N, Woodhouse M, Serrano-Pedraza I. Overestimation of stereo thresholds by the TNO stereotest is not due to global stereopsis. Ophthalmic and Physiological Optics. 2017;37(4):507-20. doi: https://doi.org/10.1111/opo.12371.
