# Supplementary material for: Design, development and pilot of a realistic virtual reality application to analyse quick directional change in sport: Avatar cutting scenario with alterable parameters
Source: PLoS One. 2025 Jun 24;20(6):e0324941. doi: 10.1371/journal.pone.0324941 (PMC12186900; doi:10.1371/journal.pone.0324941)
Supplement: S3 Protocol — (PDF) [file pone.0324941.s003.pdf]

Systems and setup

In 1 collection

Hannah K.M. Tang[1,2], Mark J. Lake[3,2], Richard J. Foster[3,2], Frederic A. Bezombes[1,2]

[1]School of Engineering, LJMU, UK;
[2]Current address: Liverpool John Moores University, Byrom St, Liverpool, L3 3AF;
[3]Research Institute for Sport and Exercise Sciences, LJMU, UK

Hannah Tang
Liverpool John Moores University

Protocol Info: Hannah K.M. Tang, Mark J. Lake, Richard J. Foster, Frederic A. Bezombes . Systems and setup. **protocols.io**
https://protocols.io/view/systems-and-setup-d7rk9m4w

Created: April 07, 2025

Last Modified: April 10, 2025

Protocol Integer ID: 126476

Keywords: Virtual reality, Avatar, Arrows, Cutting, Quick directional change, Sport, Biomechanics

Funders Acknowledgements:
Liverpool John Moores University in the form of Doctor of Philosophy funding
Grant ID: Student ID 854667

Abstract

This protocol contains the lab procedure for the PLOS ONE paper, "Lab protocol for a realistic virtual reality application to analyse quick directional change in sport: Avatar cutting scenario with alterable parameters".
This section of the protocol pertains to the experiment's systems and setup.

Safety warnings

Prior to data collection, all individuals were informed of the safety provisions. This included:

- The role of the periphery foam on the floor.
- That the primary researcher would monitor movement.
- Individuals had a minimum of one additional visual 'spotter' (other than the primary researcher) who watched the participant when moving with a headset on.
- Participants were told to emergency stop if the command 'Stop!' was yelled at any time.
- Participants were to jog or run every recorded trial at their own 'safe maximum' (the maximum speed at which they felt safe). They were asked to continually monitor this. However, individuals were prompted to slow down if their speed became a safety risk.
- Individuals rested for a minimum of 3 minutes every 8 trials. A resting seat, outside of the capture volume, was identified for their use and they were told to request a break, if needed, at any time.

Ethics statement

Liverpool John Moores University Research Ethics Committee reference: 22/ENR/004.
Participants provided written informed consent to take part in the study and to publish these case details. The research was conducted in accordance with the Declaration of Helsinki. The participants were medically screened, primarily ensuring no musculoskeletal complaints in 6 months, or issues with vision, balance, or neurological impairment.

Before start

The VR application can be downloaded from GitHub, in-line with the licence outlined on GitHub:
https://github.com/HannahKTang/VR-for-movement-assessment-in-sport.git
The VR application can be referenced with the following DOI:
https://doi.org/10.5281/zenodo.15102390

Prior to data collection

1 Two personal computers (PCs) run simultaneously: one to deliver the virtual reality (VR) scene via headset, and one for motion capture.

The VR system

2 The VR system consisted of a high-powered PC, a HTC Vive Pro head-mounted display (HTC, New Taipei City, Taiwan), with the HTC Vive Wireless Adapter attachment (attached to the top of the headset and detected by a HTC Wireless Link Box sensor connected to the computer via a peripheral component interconnect [PCI]), and six HTC lighthouses/base stations (2.0).

3 The PC required software:

- Unity game engine
- Steam VR
- HTC Vive Wireless

> Note
>
> The PC needed to be sufficient to meet the requirements of the specific headset and software.
> PC specifications in current study:
> - *Graphics card*: NVIDIA GeForce GTX970
> - *RAM*: 32.0 GB
> - *Processor*: Intel(R) Core(TM) i7-4790K CPU @ 4.00GHz
> - *System type*: 64-bit Operating System, x64-based processor

4 A spare headset (with wireless adapter) and a spare high-powered PC (with PCI Wireless Link Box) were set up in case of headset malfunction.

> Note
>
> This has not been needed.

VR application

5 The required software (Unity game engine, Steam VR, VIVE headset and VIVE Wireless) were installed using the guides at the following webpages:

- Unity game engine: https://unity.com/products/unity-personal [Accessed: 10/04/2025]
- Steam VR: https://store.steampowered.com/app/250820/SteamVR/ [Accessed: 10/04/2025]
- VIVE headset and VIVE Wireless: https://www.vive.com/uk/setup/pc-vr/ [Accessed: 10/04/2025]

6

Note

The following steps allow the user to access the VR application on the assumption that Unity game engine, Steam VR, the Vive headset and Vive Wireless were set up correctly and calibrated. Steam VR, Vive headset and Vive Wireless must be active when the Unity scene is opened. If this is not the case, the scene can still be opened; however, when playing the scene, the orientation of the camera will not be correct, and the presentation of the VR arrow on the VR TV screen or the movement of the avatar may not occur when moving through the virtual timing gates. Please reference this DOI when using the VR application downloaded from GitHub, in line with the licence outlined on GitHub: https://doi.org/10.5281/zenodo.15102390

7 Go to the following webpage: https://github.com/HannahKTang/VR-for-movement-assessment-in-sport.git [Accessed: 10/04/2025]

8 Select “Code” and “Download Zip”, see Figure 1.

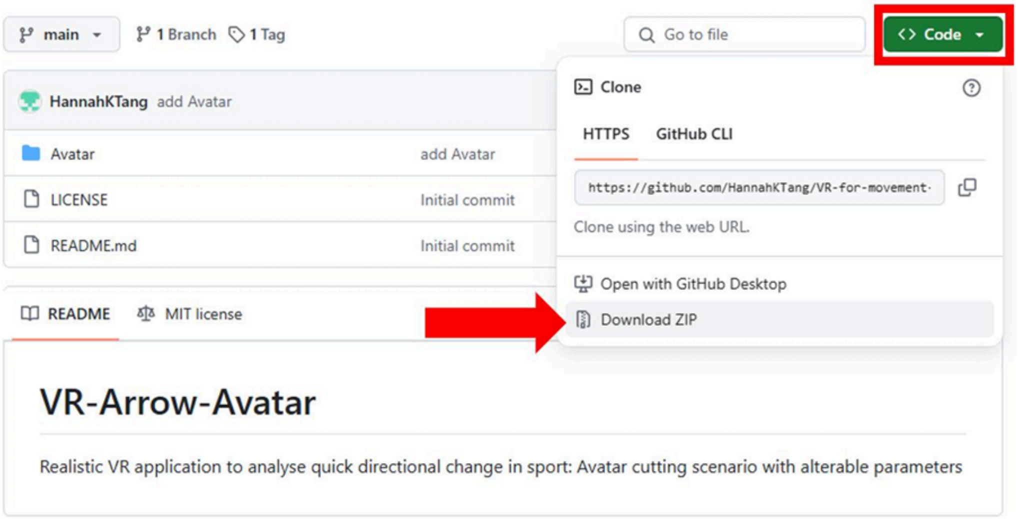

Fig. 1 Download zip file

9 Open the zip file and click and drag the unzipped downloaded folder to a local location of choice on the PC.

10 There are many methods to open the VR scene itself; the following is one method. Open the folder “Avatar”, see Figure 2.

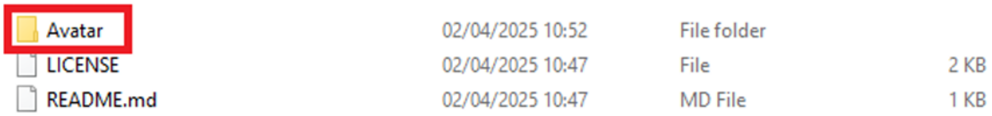

Fig. 2 Open avatar folder

11 Open the folder “Assets”, see Figure 3.

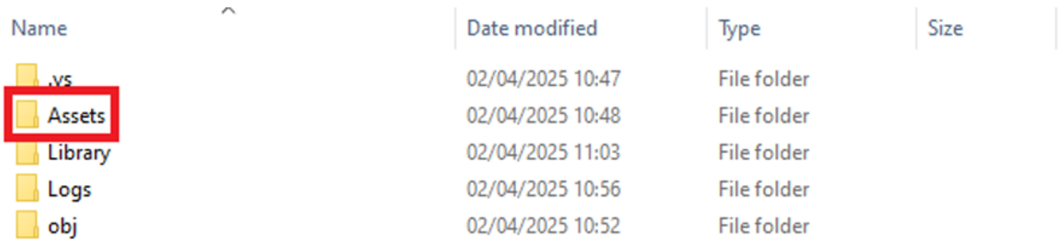

Fig. 3 Open assets folder

12 Open the file “Avatar(5)”, see Figure 4.

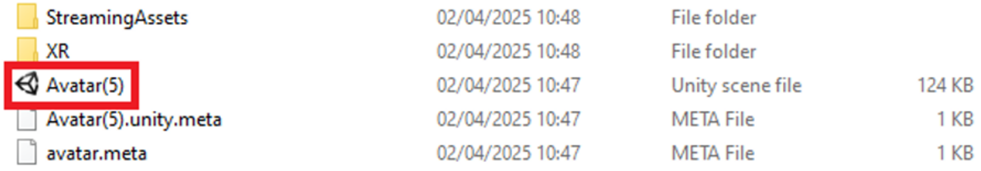

Fig. 4 Open Avatar (5) file

13 To add or remove the avatar from the scene, select “Armature left” in the Hierarchy, see Figure 5, and select this in the “Inspector”, see Figure 6.

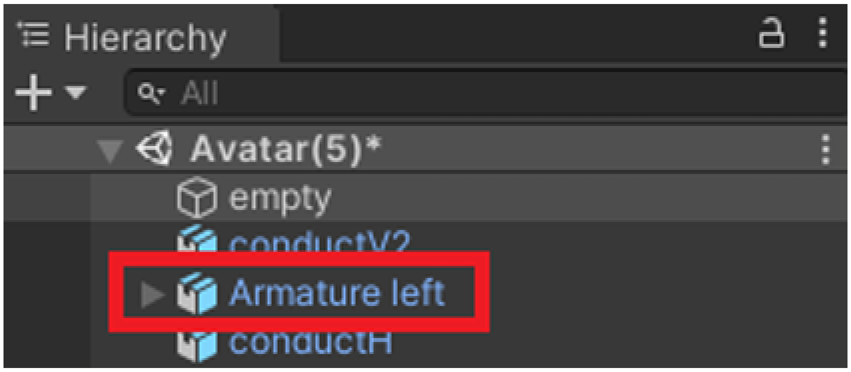

Fig. 5 Selection of avatar object

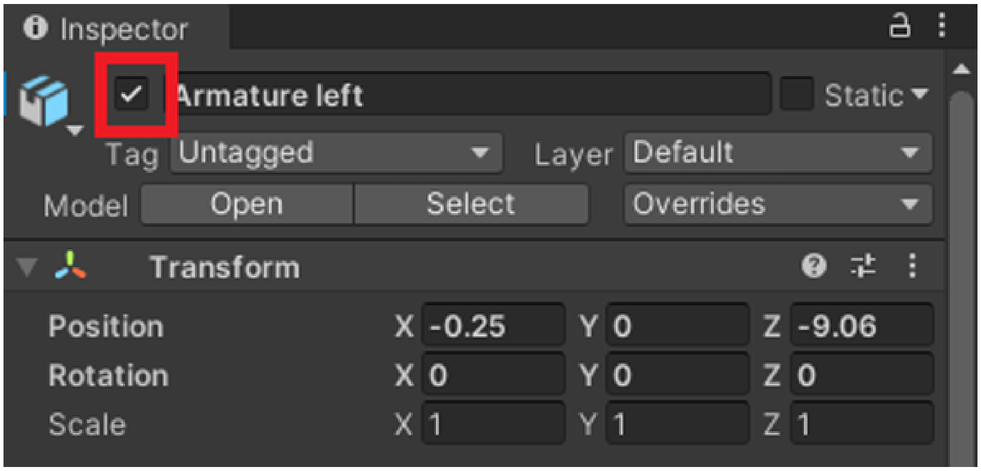

Fig. 6 Making the avatar object visual in the virtual scene

14 To operate the arrows, select “Randomise_cutting (Script)” in the Inspector, see Figure 7.

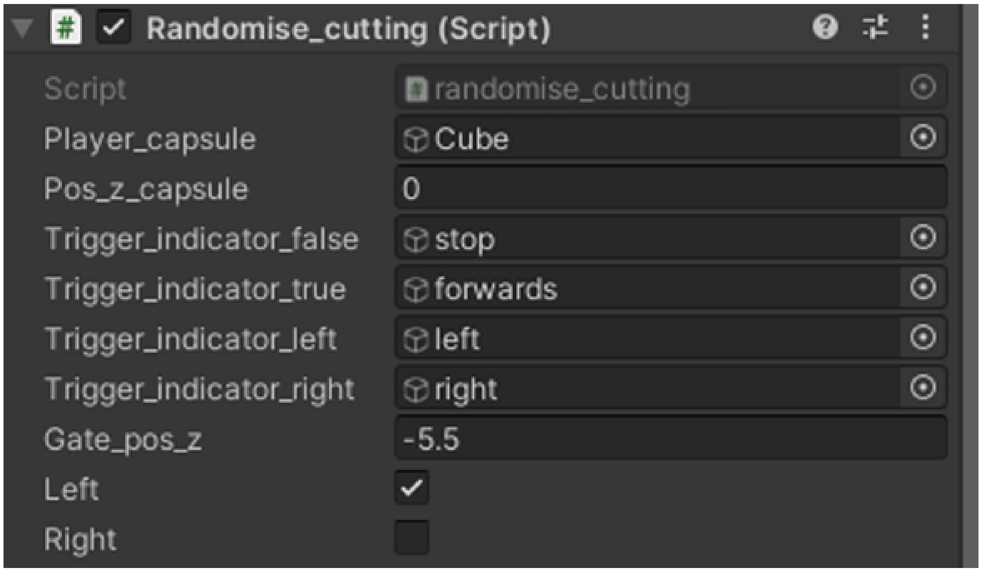

Fig. 7 Operate arrows

14.1 The following parameters can be changed in the arrow condition:

- The player capsule (currently set as a cube object)
- The gate position (Gate_pos_z)
- The direction of the arrow left or right (Figure 8)

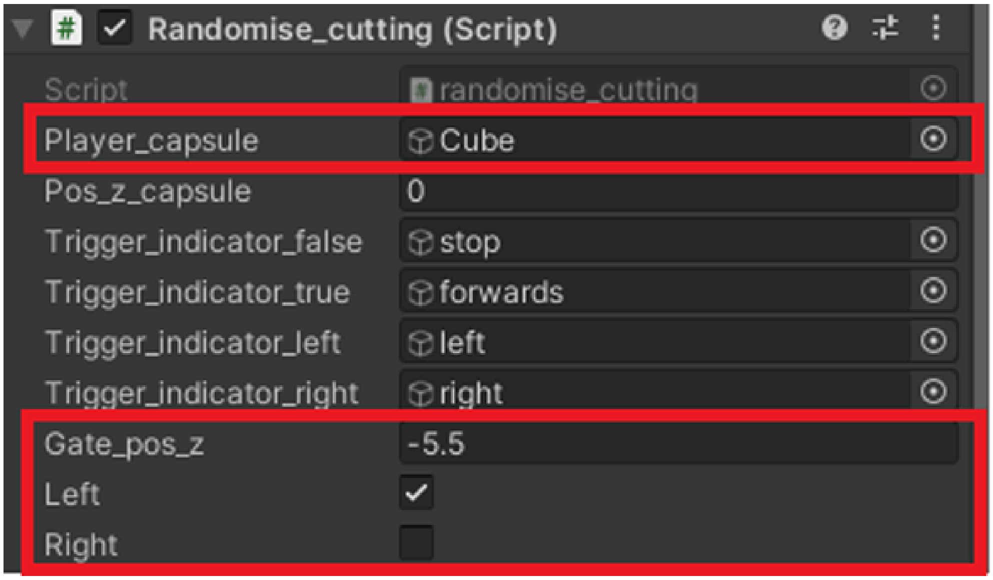

Fig. 8 Arrow parameters

15 To operate the avatar, select “Avatar_cutting (Script)” in the Inspector, see Figure 9.

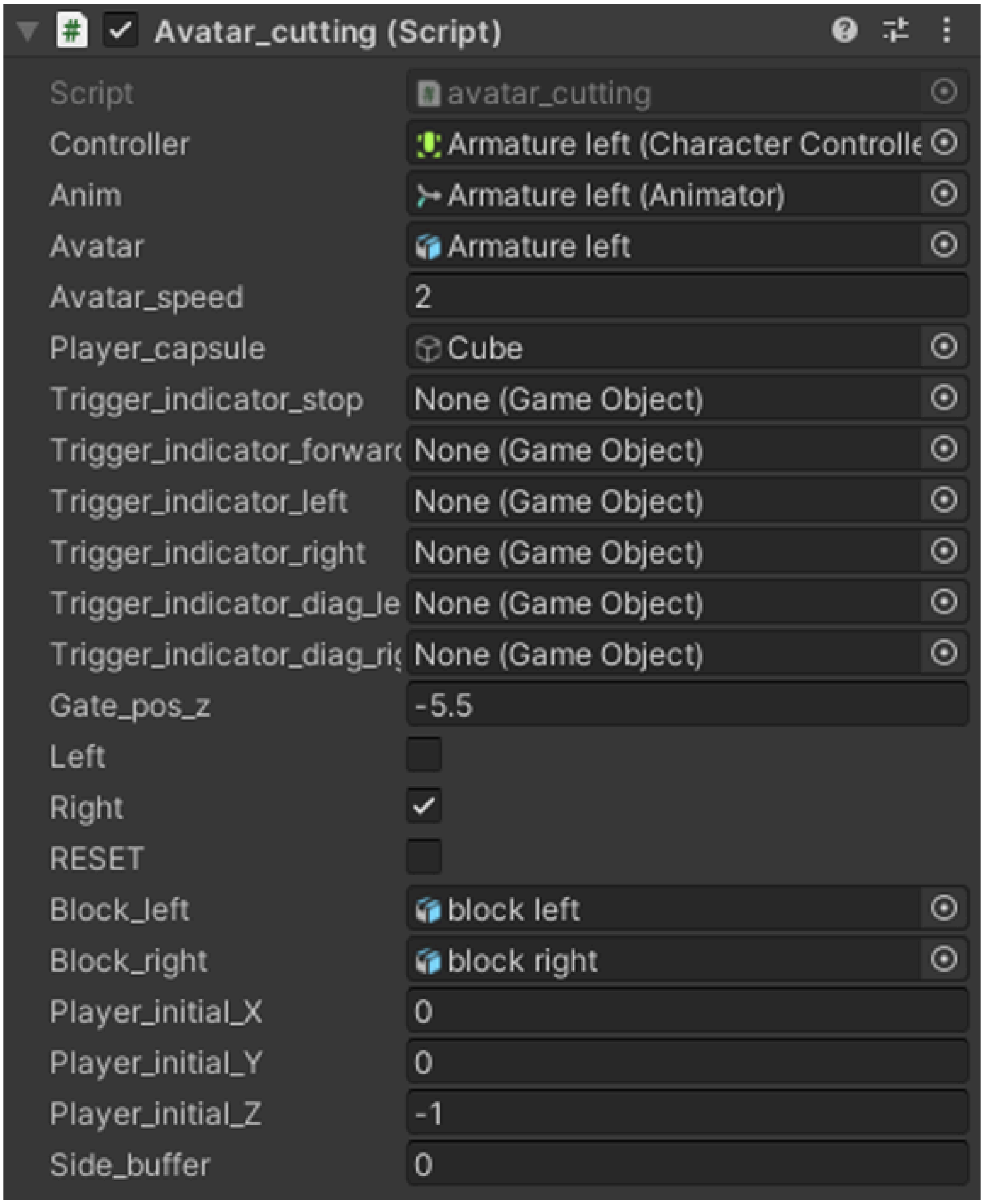

Fig. 9 Operate avatar

15.1 The following parameters can be changed in the avatar condition:

- The player capsule (currently set as a cube object)
- The gate position (Gate_pos_z)
- The player position at the start of the scene, avatar speed, and the direction of the avatar end position (left or right of the headset approach) (Figure 10)

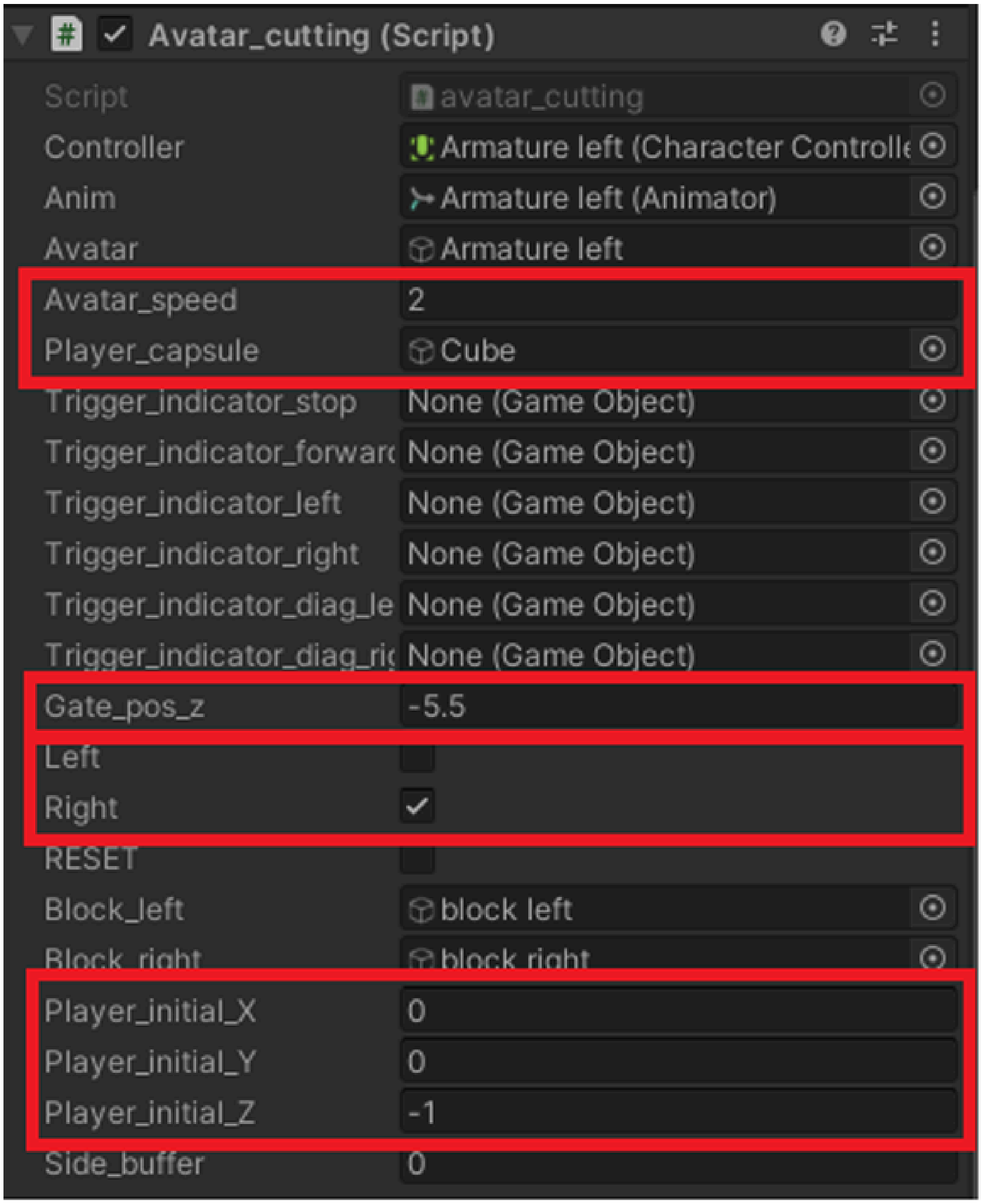

Fig. 10 Avatar parameters

Note

The player position is set relative to the room at the start of the scene, and the gate position is set relative to the player. The gate position is only moved in the z axis, towards and away from the VR TV screen and avatar.

16 To begin the movement in the VR environment, press play (Figure 11) and move towards the VR TV screen or avatar.

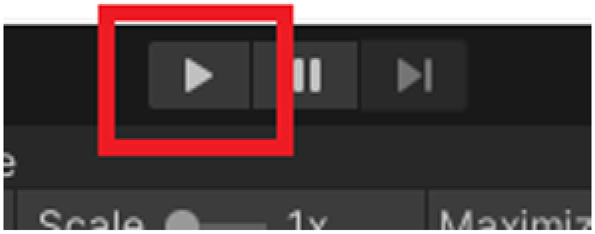

Fig. 11 To begin the movement in the VR environment

The motion capture system

17 The motion capture system consisted of a PC and 14 infra-red motion capture cameras (Qualisys, Gothenburg, Sweden; model: Arqus A12).

The PC required motion capture software:

- Qualisys Track Manager

> **Note**
>
> Cameras were set at varying heights to capture whole-body data.
> See Figure 12 for relative positions of cameras. This was set to collect whole-body kinematic data at a frequency of 250 Hz.

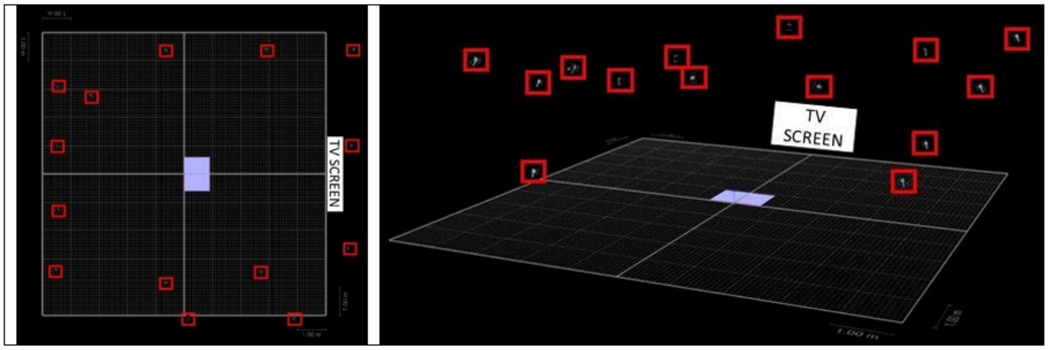

Fig. 12 Relative position of cameras

Experimental set-up

18 Each of the three conditions required the preparation and delivery of different visual stimuli to direct movement (Table 1).

Table 1. Three conditions of the study

A	B
Conditions:	Environment and task:
1 – Non-VR Arrows	In the physical world, three stimuli were presented on a TV screen (110 cm): a cross sign for stop, a left arrow for cut left, or a right arrow for cut right.
2 – VR Arrows	In a VR emulation of the data collection area, three stimuli were presented on a direct VR replication of the physical TV screen: a cross sign for stop, a left arrow for cut left, or a right arrow for cut right.
3 – VR Avatar	In the same virtual emulation of the biomechanics laboratory, individuals reacted by moving in the opposite direction to the blocking manoeuvre of a VR avatar that approached and cut 30° left or right of approach.

19 Experimental set-up in the physical world was directly emulated as a virtual environment (Figure 13).

Note

The present study and VR environment were based on a typical laboratory-based cutting assessment.

- Electrical tape was placed to mark the starting position 3.3 m before the edge of two 60 × 90 cm force plates.
- Timing gates (Brower TCi system, UT, US) were positioned 1 m from the starting position and 1 m from each other.
- Each visual stimulus was presented as the headset user moved through the second timing gate (physical or virtual).

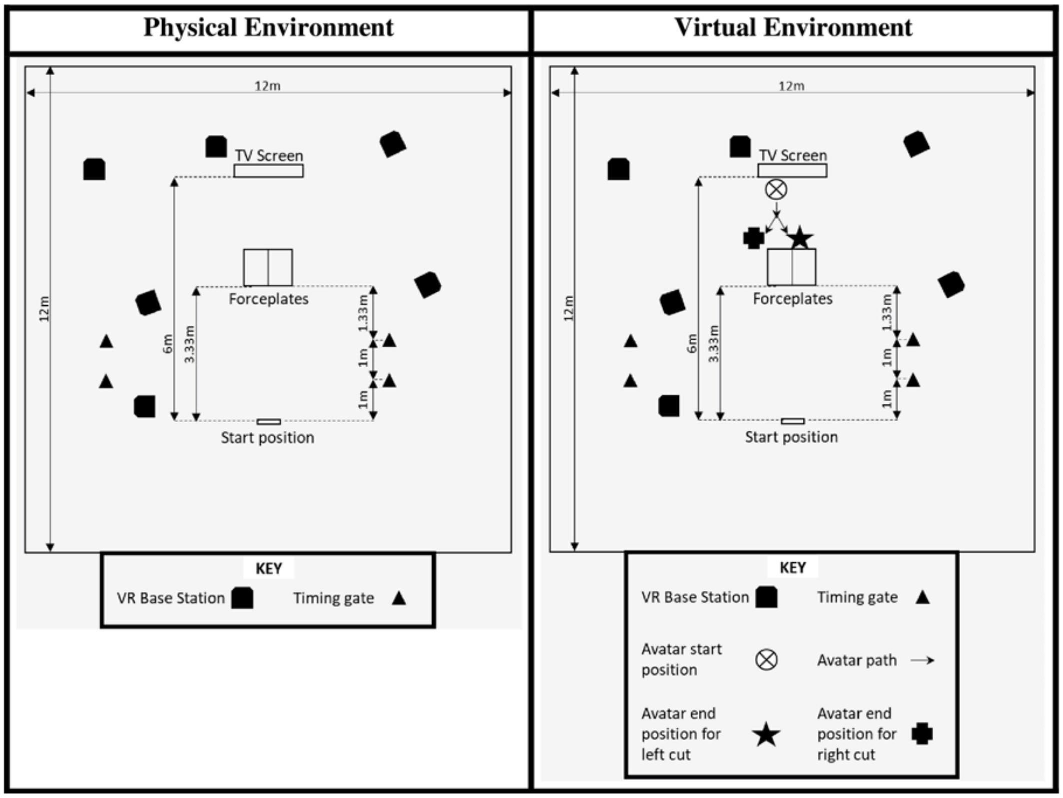

Fig. 13 Experimental parameters in the physical and virtual environment

Initial correspondence

20 Initial correspondence via email ensured:

1. Participants were emailed a general overview of the study purpose and what it would involve.
2. Participants were provided with an electronic copy of the information sheet (S2) and informed consent
   document (S3) to decide whether to participate. If taking part, this was provided in hardcopy on the day of data collection.
3. A data collection time and location were arranged.
4. Participants were informed of suitable motion-capture attire (tight fitting and not retroreflective).

Note

Participants completed the study in their own footwear to maintain ecological validity and to ensure familiarity with footwear to reduce risk factors.

Day of data collection prior to participant arrival: Calibration

21 Both the VR and motion capture systems require calibration prior to each data collection session.

21.1 **VR calibration** involved setting the room-scale play area based on a perimeter, which was a minimum of 1.5 m from all objects, and was created by thin foam sheets on the floor. In the VR environment, the safety boundary for the play area was not visible unless it was breached.

21.2 **Motion capture calibration** involved a calibrated volume that covered all areas in which movement data was collected. See Figure 14 for example of calibrated volume.

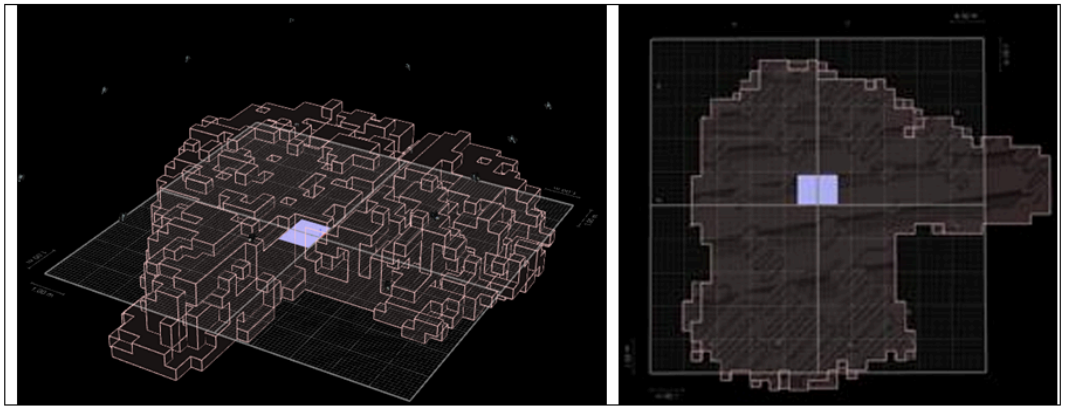

Fig. 14 Example of the calibrated volume
