# Supplementary material for: Design, development and pilot of a realistic virtual reality application to analyse quick directional change in sport: Avatar cutting scenario with alterable parameters
Source: PLoS One. 2025 Jun 24;20(6):e0324941. doi: 10.1371/journal.pone.0324941 (PMC12186900; doi:10.1371/journal.pone.0324941)
Supplement: S4 Protocol — (PDF) [file pone.0324941.s004.pdf]

Data collection protocol: Screening and motion trial preparation

In 1 collection

Hannah K.M. Tang[1,2], Mark J. Lake[3,2], Richard J. Foster[3,2], Frederic A. Bezombes[1,2]

[1]School of Engineering, LJMU, UK;
[2]Current address: Liverpool John Moores University, Byrom St, Liverpool, L3 3AF;
[3]Research Institute for Sport and Exercise Sciences, LJMU, UK

Hannah Tang
Liverpool John Moores University

Protocol Info: Hannah K.M. Tang, Mark J. Lake, Richard J. Foster, Frederic A. Bezombes . Data collection protocol: Screening and motion trial preparation. **protocols.io** **https://protocols.io/view/data-collection-protocol-screening-and-motion-tria-d7rm9m46**

Created: April 07, 2025

Last Modified: April 10, 2025

Protocol Integer ID: 126477

Keywords: Virtual reality, Avatar, Arrows, cutting, Quick directional change, Sport, Biomechanics

Funders Acknowledgements:
Liverpool John Moores University in the form of Doctor of Philosophy funding
Grant ID: Student ID 854667

Abstract

This protocol contains the lab procedure for the PLOS ONE paper, "Lab protocol for a realistic virtual reality application to analyse quick directional change in sport: Avatar cutting scenario with alterable parameters". This section of the protocol pertains to the participant screening and motion trial preparation during data collection.

Safety warnings

Prior to data collection, all individuals were informed of the safety provisions. This included:

- The role of the periphery foam on the floor.
- That the primary researcher would monitor movement.
- Individuals had a minimum of one additional visual 'spotter' (other than the primary researcher) who watched the participant when moving with a headset on.
- Participants were told to emergency stop if the command 'Stop!' was yelled at any time.
- Participants were to jog or run every recorded trial at their own 'safe maximum' (the maximum speed at which they felt safe). They were asked to continually monitor this. However, individuals were prompted to slow down if their speed became a safety risk.
- Individuals rested for a minimum of 3 minutes every 8 trials. A resting seat, outside of the capture volume, was identified for their use and they were told to request a break, if needed, at any time.

Ethics statement

Liverpool John Moores University Research Ethics Committee reference: 22/ENR/004.
Participants provided written informed consent to take part in the study and to publish these case details. The research was conducted in accordance with the Declaration of Helsinki. The participants were medically screened, primarily ensuring no musculoskeletal complaints in 6 months, or issues with vision, balance, or neurological impairment.

Before start

The VR application can be downloaded from GitHub, in-line with the licence outlined on GitHub:
https://github.com/HannahKTang/VR-for-movement-assessment-in-sport.git
The VR application can be referenced with the following DOI:
https://doi.org/10.5281/zenodo.15102390

Data collection

1 On participant arrival, participants were provided with two hard copies of the information sheet (S2) and informed consent document (S3).

Note

One hard copy was for participants to retain and one for the researcher.

2 Participants were medically screened prior to data collection, with a medical questionnaire (S3).

3 Vision and visual judgement were assessed in three ways:

1. Visual acuity
2. Contrast sensitivity
3. Depth perception

4 **Visual acuity and contrast sensitivity** were assessed using The Freiburg Visual Acuity Test (Figure 15, 16), which uses an automated protocol for presenting single optotypes, accessible at FrACT: https://michaelbach.de/fract/ [Accessed: 10/04/2025].

Note

In this instance, Landolt rings were presented with eight possible gap directions [1, 2].

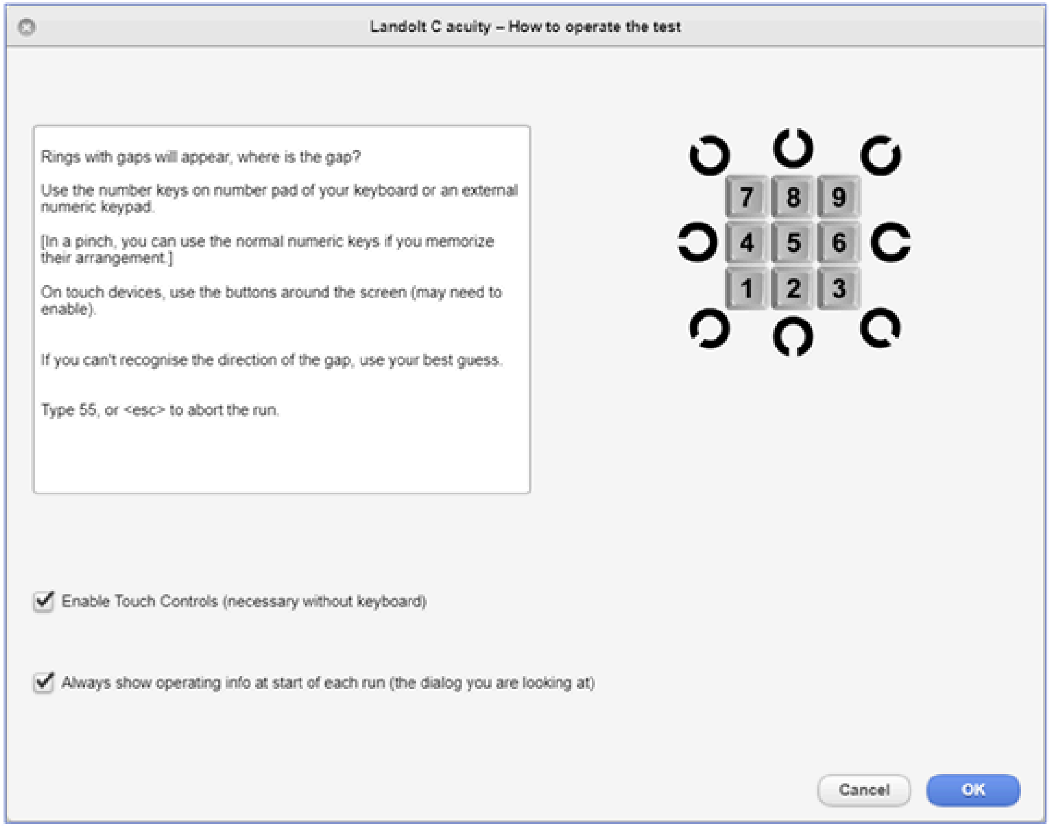

Fig. 15 The Freiburg Visual Acuity Test operating instructions

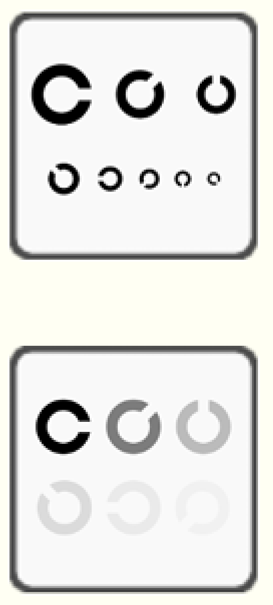

Fig. 16 The Freiburg Visual Acuity Test, example of acuity test (above) and contrast tests (below)

For visual acuity, participants were excluded if scores were 0.40 Log Mar or higher (equating to 20/50) vision on the Snellen (ft) scale, indicating mild impairment or worse.

Note

See WHO blindness and vision impairment guide: https://www.who.int/news-room/fact-sheets/detail/blindness-and-visual-impairment [Accessed: 10/04/2025]).
See the following link for test values and meanings:
https://michaelbach.de/sci/acuity.html [Accessed: 10/04/2025].

For contrast sensitivity, participants were excluded if scores were equal to or lower than 1.5 Log CS [3].

Note

Scores of less than 1.5 are consistent with visual impairment [3].

5 **Depth perception** was assessed using the Stereovision Stereo Fly Test (Figure 17) with polarised lenses. This involved four circles arranged in a diamond configuration.
The Stereovision Stereo Fly Test (Stereo Optical, Chicago) can be accessed at:
https://www.stereooptical.com/products/stereotests-color-tests/original-stereo-fly/
[Accessed: 10/04/2025].

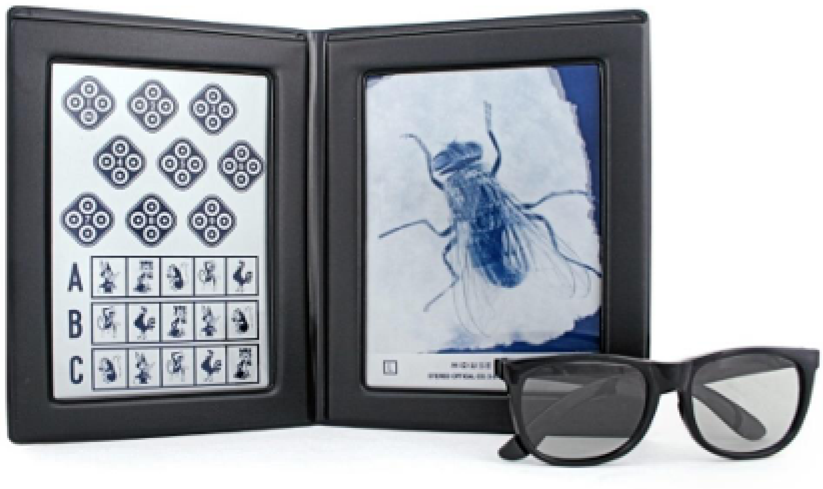

Fig. 17 Stereovision Stereo Fly Test equipment

Participants identified one of the four circles that appeared to have a different visual depth to the other three. There were nine sets in total.

Note

For the Stereovision Stereo Fly Test manual, see: https://www.stereooptical.com/wp-content/uploads/2018/01/LEA-symbols-FLY-2017-User-manual-ONLY-12212017-2.pdf [Accessed: 10/04/2025].

Depth perception was recorded in order to report the range of discrepancy between participants.

Note

For this assessment it was difficult to apply a threshold, as there is disparity between stereopsis tests [4]. However, the assessment can still be used to identify any outliers and the range in depth perception of participants.

6 Participant mass (kg) and height (cm) were measured.

7 A marker model was applied. A six degree of freedom (6DOF) marker model set was used to identify each segment independently, comprising a total of 66 reflective markers (see Figure 18 and Table 2 for marker model set).

Note

This marker model set is comprehensive, and markers labelled for skeletal modelling can be selected post data collection depending on requirements.

To model segments of the body with 6DOF, segment defining markers were placed on: the head, shoulders, trunk, elbows, wrists, hands, pelvis, thighs, shanks, ankles and shoes. Additionally, the upper limb segments of the upper arm and forearm had one additional tracking marker, and four-marker clusters were placed on each lower limb segment of the thighs and shanks. The head markers comprised 6 markers either on a headband or the VR headset (not placed on the HTC Vive movable headphones which remained in a raised position to not impede verbal instructions to the wearer).

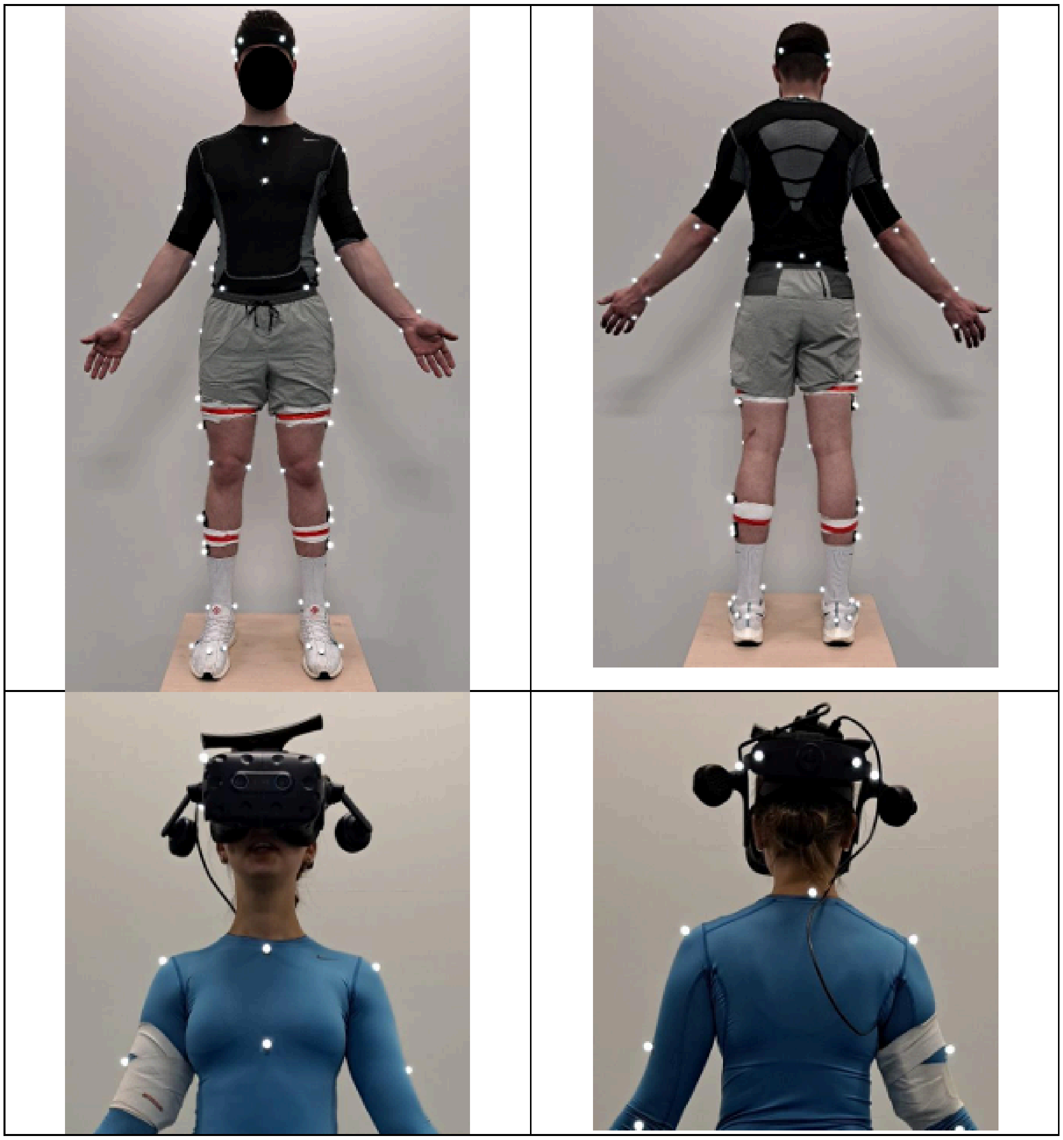

Fig. 18 Marker model set with headband (top) and virtual reality headset (bottom)

Note

Medial knee and ankle markers were removed for data collection to allow for unhindered movement.

Table 2. Marker model set

A	B
	**Label**
Upper body	
6	Head band/Set
2	Acromion process

A	B
1	Tubular notch
1	Styloid process
1	T1
1	T12
2	Humerus lateral epicondyle
2	Humerus medial epicondyle
2	Styloid process of radius
2	Styloid process of ulna
2	Head of 5th metacarpal
2	Upper arm
2	Forearm
Pelvis/hip	
2	Iliac Spine
2	Anterior Superior Iliac Spine
2	Posterior Superior Iliac Spine
2	Greater trochanter
Upper leg	
8 (4 per cluster)	Thigh cluster
2	Femoral lateral epicondyle
2	Femoral medial epicondyle
Lower leg/ankle	
8 (4 per cluster)	Shank clusters
2	Lateral malleolus
2	Medial malleolus
2	1st distal metatarsal head
2	2nd proximal interphalangeal joint
2	5th distal metatarsal head
2	Heel
2	Outer foot

Note

If the alternative headset was used, or any marker displaced on the body during data collection, a new standing calibration file would have been taken for the corresponding motion trials.

Protocol references

1. Bach M. The Freiburg Visual Acuity Test-automatic measurement of visual acuity. Optometry and vision science. 1996;73(1):49-53.

2. Bach M. The Freiburg Visual Acuity Test-variability unchanged by post-hoc re-analysis. Graefe's Archive for
Clinical and Experimental Ophthalmology. 2006;245:965-71. doi: https://doi.org/10.1007/s00417-006-0474-4.

3. Parede TRR, Torricelli AAM, Mukai A, Vieira Netto M, Bechara SJ. Quality of vision in refractive and cataract
surgery, indirect measurers. Arquivos Brasileiros de Oftalmologia. 2013;76:386-90. doi: https://doi.org/10.1590/S0004-27492013000600016.

4. Vancleef K, Read JC, Herbert W, Goodship N, Woodhouse M, Serrano-Pedraza I. Overestimation of stereo thresholds by the TNO stereotest is not due to global stereopsis. Ophthalmic and Physiological Optics. 2017;37(4):507-20. doi: https://doi.org/10.1111/opo.12371.
