# Supplementary material for: Design, development and pilot of a realistic virtual reality application to analyse quick directional change in sport: Avatar cutting scenario with alterable parameters
Source: PLoS One. 2025 Jun 24;20(6):e0324941. doi: 10.1371/journal.pone.0324941 (PMC12186900; doi:10.1371/journal.pone.0324941)
Supplement: S5 Protocol — (PDF) [file pone.0324941.s005.pdf]

Data collection protocol: Motion trials

In 1 collection

Hannah K.M. Tang[1,2], Mark J. Lake[3,2], Richard J. Foster[3,2], Frederic A. Bezombes[1,2]

[1]School of Engineering, LJMU, UK;
[2]Current address: Liverpool John Moores University, Byrom St, Liverpool, L3 3AF;
[3]Research Institute for Sport and Exercise Sciences, LJMU, UK

Hannah Tang
Liverpool John Moores University

Protocol Info: Hannah K.M. Tang, Mark J. Lake, Richard J. Foster, Frederic A. Bezombes . Data collection protocol: Motion trials. **protocols.io https://protocols.io/view/data-collection-protocol-motion-trials-d7rn9m5e**

Created: April 07, 2025

Last Modified: April 10, 2025

Protocol Integer ID: 126478

Keywords: Virtual reality, Avatar, Arrows, Cutting, Quick directional change, Sport, Biomechanics

Funders Acknowledgements:
Liverpool John Moores University in the form of Doctor of Philosophy funding
Grant ID: Student ID 854667

Abstract

This protocol contains the lab procedure for the PLOS ONE paper, "Lab protocol for a realistic virtual reality application to analyse quick directional change in sport: Avatar cutting scenario with alterable parameters".
This section of the protocol pertains to the motion trials that occur during data collection.

Safety warnings

Prior to data collection, all individuals were informed of the safety provisions. This included:

- The role of the periphery foam on the floor.
- That the primary researcher would monitor movement.
- Individuals had a minimum of one additional visual 'spotter' (other than the primary researcher) who watched the participant when moving with a headset on.
- Participants were told to emergency stop if the command 'Stop!' was yelled at any time.
- Participants were to jog or run every recorded trial at their own 'safe maximum' (the maximum speed at which they felt safe). They were asked to continually monitor this. However, individuals were prompted to slow down if their speed became a safety risk.
- Individuals rested for a minimum of 3 minutes every 8 trials. A resting seat, outside of the capture volume, was identified for their use and they were told to request a break, if needed, at any time.

Ethics statement

Liverpool John Moores University Research Ethics Committee reference: 22/ENR/004.
Participants provided written informed consent to take part in the study and to publish these case details. The research was conducted in accordance with the Declaration of Helsinki. The participants were medically screened, primarily ensuring no musculoskeletal complaints in 6 months, or issues with vision, balance, or neurological impairment.

Before start

The VR application can be downloaded from GitHub, in-line with the licence outlined on GitHub:
https://github.com/HannahKTang/VR-for-movement-assessment-in-sport.git
The VR application can be referenced with the following DOI:
https://doi.org/10.5281/zenodo.15102390

Motion trial

1 Prior to data collection, all individuals were informed of the safety provisions. This included:

- The role of the periphery foam on the floor.
- That the primary researcher would monitor movement.
- Individuals had a minimum of one additional visual ‘spotter’ (other than the primary researcher)
  who watched the participant when moving with a headset on.
- Participants were told to emergency stop if the command ‘Stop!’ was yelled at any time.
- Participants were to jog or run every recorded trial at their own ‘safe maximum’ (the maximum
  speed at which they felt safe). They were asked to continually monitor this. However, individuals were prompted to slow down if their speed became a safety risk.
- Individuals rested for a minimum of 3 minutes every 8 trials. A resting seat, outside of the capture
  volume, was identified for their use and they were told to request a break, if needed, at any time.

> **Note**
>
> A written script, specific to the study design, ensured uniform delivery of information before each block trial.

2 The 3 conditions were explained - that each condition would use a different visual cue to cut:

1. Physical world with arrows
2. VR emulation of the physical world with VR arrows
3. VR emulation of the physical world with an avatar opponent displaying a blocking manoeuvre

Participants were told to cut in the opposite direction to the avatar's movement.

3 The three movements to be captured over the data collection session were demonstrated:

1. An immediate stop
2. a 90-degree cut to the left
3. a 90-degree cut to the right

4 Participants stood in the standard anatomical position for a standing calibration, upon which the skeleton would be modelled for the corresponding motion trials.

5 Individuals were provided with incremented familiarisation tasks. Familiarisation was repeated in four stages at the beginning of each condition:

1. Walk forward and stop at presentation of stimulus
2. Jog forward and stop at presentation of stimulus
3. Walk entire manoeuvre
4. Jog entire manoeuvre

This was repeated for a stop sign, cut left and cut right.

Note

Individuals were allowed to repeat this familiarisation process for each condition for as long as they needed until they felt safe.

Practice and warm-up time was not capped, and individuals were asked to practice until able to perform a cutting manoeuvre with the following criteria:

1. The direction of approach was in a straight line towards the television screen
2. A cutting leg was planted (the opposite leg to the direction of cut)
3. The individual moved perpendicularly to the direction of approach (not in an arc)
4. The manoeuvre was a side-step cut, not a different form of cut (e.g. shuffle-step cut)
5. The individual completed at least two follow-through steps

Participants were asked to continue until they conducted at least two successful practice trials in each condition.

6 The starting view of all environments and direction of movement was maintained. The Unity scene was launched before the data capture commenced on Qualysis Track Manager and before the motion trial began. To increase realism, the avatar swayed in their starting position until the avatar's movement was instigated.

Note

In both the physical and virtual world, individuals began each trial facing the same direction with the same perspective on the laboratory. Therefore, their direction of movement had them pass through physical, or physical and virtual, timing gates towards a pair of force plates, as well as towards the television screen, and in the third condition, the avatar.

7 Data collection continued until a minimum of 5 left motion trials were successfully collected.

Note

The ratio of left 90-degree cuts, to stop and right cuts, was 3:1. This order of stimulus presentation was randomised, both block randomised by trial number within each condition, and then again randomised by order of image being presented within each condition.

8 Individuals rested for a minimum of 3 minutes every 8 trials.

9 Approach speed was measured for each motion trial, and this was then later calculated again during motion trial analysis for validation.

10 At the end of the data collection session, an average of three walking trials at self-selected walking speed and three maximum running speeds (all without headset) were taken to act as a baseline speed for comparison.

11 System-related events were noted throughout the use of the VR application. Events were noted if they involved any irregularities, any limitations or issues with headsets or the application, and any factors that were not problematic but might influence the use of the application in the future.

Note

Stage **4-11** were repeated in the VR conditions to the cue of: 1) the VR arrows, and 2) the VR avatar opponent. go to step #4 and repeat for each condition.

When the VR headset and wireless adapter were worn, the total weight of the headset and wireless adapter, with corresponding cables to the battery pack, was approximately 1 kg. The battery pack itself (approximately 220g) was bound to the dominant arm using two elasticated and Velcro medical wraps, secured with electrical tape. The cable connecting the battery pack to the wireless adapter attachment was of sufficient length to allow for free ambulatory movement, so the arms, trunk, and head were not restricted, and was positioned using the user's clothing (tucking in if suitable) so as to not obstruct the view of the retroreflective markers. Additionally, a minimum of one additional 'spotter' watched the participant when moving with a headset on. The VR scene commenced prior to the motion being captured. Before moving through the timing gates, the virtual TV remained black, and the avatar swayed on the spot. After each motion trial, on return to the start position, participants were asked to raise the headset minimally and were asked to minimise the amount of time spent looking at the back of the room (maintaining the connection of the Wireless Link Box).

12 After the motion trials, participants answered an eight-question questionnaire (Table 3). This determined any disparities or impacts of: dominant leg use, prior participation in biomechanical research or sport, details of prior VR use, and perspectives on the experience of using the VR environment.

Table 3. Eight-question questionnaire to gain participant information

A
1. Which is your dominant leg?
2. Have you participated in a study that has measured quick directional change (cutting) trials before?
3. Have you ever competed in a sport (at any level) where you would do a movement like this? If yes, which and at what level?
4. How many hours would you normally spend using a VR headset in any given week?
5. Did you feel like the virtual environment (virtual lab room) was similar and different from the real environment? If yes, how?
6. Do you feel like you moved differently in the virtual environment compared to the real environment? If yes, how?
7. Did you feel like there was a difference between how the arrows directed your movement compared to the avatar? If yes, how?
8. Is there anything else you feel would improve the VR experience either as a user, or for sports, or for rehabilitation?

13 Participants completed a Likert-based System Usability Scale of 24 comments (Table 4).

Table 4. Likert-based System Usability Scale of 24 comments

A	B	C	D	E	F	G
System Usability Scale		Strongly Disagree				
		1	2	3	4	5
1	I would use the system again					
2	I thought that the virtual room looked similar to the physical room					
3	I thought the virtual room felt similar to the physical room					
4	The objects in the virtual room looked the same scale as they do in the physical room					
5	The objects in the virtual room appeared to be in the same/similar location as they did in the physical room					
6	The virtual room did not make me feel sick					
7	I felt confident walking in the physical room					

A	B	C	D	E	F	G
8	I felt confident walking in the virtual room					
9	I felt confident walking with the headset on my head					
10	I felt confident jogging in the physical room					
11	I felt confident jogging in the virtual room					
12	I felt confident jogging with the headset on my head					
13	I felt able to change direction in the physical room					
14	I felt able to change direction in the virtual room					
15	I felt able to change direction with the headset on my head					
16	I think if I were in a bigger space with no obstacles, I would feel more confident walking, jogging or running in the virtual room					
17	I felt I moved in a similar way in the virtual room and the physical room					
18	I thought the arrows were similar to those in the physical world					
19	Moving around avatars felt more like a real-life situation compared to the arrows					
20	I think the avatars looked similar enough to people that I moved around them like people					
21	I do not think it matters what the avatars looked like. I would interact with them in the same way irrespective of how they look					
22	I found the decision to change direction harder with the avatar as opposed to the arrows					
23	I felt like I looked at the avatars for longer in order to make a decision to change direction as opposed to the arrows					
24	I felt like I took longer to make the decision to change direction with the avatar as opposed to the arrows					
