# Supplementary material for: Design, development and pilot of a realistic virtual reality application to analyse quick directional change in sport: Avatar cutting scenario with alterable parameters
Source: PLoS One. 2025 Jun 24;20(6):e0324941. doi: 10.1371/journal.pone.0324941 (PMC12186900; doi:10.1371/journal.pone.0324941)
Supplement: S6 Table — (PDF) [file pone.0324941.s006.pdf]

S6 Table. Issues raised and solutions taken during system optimisation

Participant	'System Usability Scale'	Discussion and notes on user and system-related events	Questionnaire	Action taken for optimisation
1	Objects in the virtual room did not look the same scale as the physical room.	Participant appeared to travel less distance in VR.<br>Forward movement in physical world did not lead to the same distance travelled in VR. Participant stated the headset felt like it was rushing in towards the avatar as the avatar began to move.	'The scale felt off.' 'I moved slower in VR than in physical world.'	Camera height and player properties were altered until run up in the physical world led to the same distance travelled in the VR environment.
2	Objects in the virtual room did not look in the same position as the physical room.	Participant kept cutting past the force plates.<br>Participant needed to be stopped on several occasions before they reached the perimeter of the VR environment and near physical objects.	'I felt as if I started further back in the VR environment.' 'It needed to be in a bigger space.'	The optimal starting position was selected in the physical world and emulated to the exact relative distance in the VR world. The headset was recentred within the mesh of the room so the user was automatically positioned on start and did not need to be recalculated on every start.
3	Virtual room made them feel sick.	Participant stated there was a constant tilt to the virtual environment, both in the frontal plane (as if the head was tilted to the side) and transverse plane (as if moving to the side of the physical TV instead of directly towards it). The VR world did look at an angle on Unity in both ways.	'Object size and positions were very similar.'<br>'VR arrows were easy to process. The avatar was much harder to evaluate.'	Placement of the headset position on the head needed to be standardised to ensure hair did not displace the headset at an angle. A short demonstration was created for each participant.<br>Calibration of the VR environment to align the virtual room with the physical room to match optimally was repeated until the process lead to an exact overlay.
