# Supplementary material for: Design, development and pilot of a realistic virtual reality application to analyse quick directional change in sport: Avatar cutting scenario with alterable parameters
Source: PLoS One. 2025 Jun 24;20(6):e0324941. doi: 10.1371/journal.pone.0324941 (PMC12186900; doi:10.1371/journal.pone.0324941)
Supplement: S7 Table — (PDF) [file pone.0324941.s007.pdf]

S7 Table. System usability scale

		Strongly disagree 1	2	3	4	Strongly agree 5
1	I would use the system again					
2	I thought that the virtual room looked similar to the physical room					
3	I thought the virtual room felt similar to the physical room					
4	The objects in the virtual room looked the same scale as they do in the physical room					
5	The objects in the virtual room appeared to be in the same/similar location as they did in the physical room					
6	The virtual room did not make me feel sick					
7	I felt confident walking in the physical room					
8	I felt confident walking in the virtual room					
9	I felt confident walking with the headset on my head					
10	I felt confident jogging in the physical room					
11	I felt confident jogging in the virtual room					
12	I felt confident jogging with the headset on my head					
13	I felt able to change direction in the physical room					
14	I felt able to change direction in the virtual room					
15	I felt able to change direction with the headset on my head					
16	I think if I were in a bigger space with no obstacles, I would feel more confident walking, jogging or running in the virtual room					
17	I felt I moved in a similar way in the virtual room and the physical room					
18	I thought the arrows were similar to those in the physical world					
19	Moving around avatars felt more like a real-life situation compared to the arrows					
20	I think the avatars looked similar enough to people that I moved around them like people					
21	I do not think it matters what the avatars looked like. I would interact with them in the same way irrespective of how they look					
22	I found the decision to change direction harder with the avatar as opposed to the arrows					
23	I felt like I looked at the avatars for longer in order to make a decision to change direction as opposed to the arrows					
24	I felt like I took longer to make the decision to change direction with the avatar as opposed to the arrows					
